# Genetic Diversity Analysis of 11 *Macrobrachium rosenbergii* Germplasms Based on Microsatellite Markers

**DOI:** 10.3390/ani16020270

**Published:** 2026-01-15

**Authors:** Tianhui Jiao, Yakun Wang, Jie Wei, Sikai Xu, Qiaoyan Zhou, Qiyao Su, Bai Liufu, Zhuang Mai, Kunhao Hong, Yayi Huang, Zikang Tu, Xidong Mu, Lingyun Yu

**Affiliations:** 1 College of Fisheries and Life Science, Shanghai Ocean University, Shanghai 201306, China; 2 Key Laboratory of Tropical and Subtropical Fishery Resources Application and Cultivation, Ministry of Agriculture and Rural Affairs, Pearl River Fisheries Research Institute, Chinese Academy of Fishery Sciences, Guangzhou 510380, Chinamuxd@prfri.ac.cn (X.M.)

**Keywords:** aquaculture, broodstock, genetic structure, genetic differentiation, giant river prawn, inbreeding

## Abstract

*Macrobrachium rosenbergii* is one of the largest and most economically significant freshwater prawns worldwide. Understanding its population genetic structure is essential for optimizing cross-breeding strategies, conserving germplasm resources, and supporting sustainable aquaculture. However, progress in this area has been hindered by the limited availability of reliable molecular markers. In this study, we developed 20 polymorphic microsatellite primer pairs and applied them to assess the genetic diversity of 11 populations collected from China and Southeast Asia (including Jiangsu, Zhejiang, Taiwan, Myanmar, Bangladesh, Sri Lanka, and Thailand). All loci exhibited high levels of polymorphism. The number of alleles (*Na*) ranged from 5 to 27, while the mean observed heterozygosity (*Ho*), expected heterozygosity (*He*), and polymorphism information content (*PIC*) were 0.570, 0.720, and 0.686, respectively. The genetic differentiation coefficient (*Fst*) among populations ranged from 0.017 to 0.289. UPGMA clustering revealed that the Myanmar population formed an independent branch, whereas the remaining ten populations clustered together, indicating relatively close genetic relationships among them. Beyond enriching the currently limited molecular marker resources for *M. rosenbergii*, this study provides essential baseline data for evaluating genetic diversity in existing populations and establishes a solid molecular foundation for future genetic monitoring and breeding programs.

## Simple Summary

Examination of newly developed genetic markers of *Macrobrachium rosenbergii* showed low genetic diversity in Chinese populations as a whole, raising concerns about the broodstock sustainability, while some Southeast Asian populations remain more distinct. These findings highlighted the urgent need to protect and better manage prawn germplasm introducing diverse populations into broodstocks.

## 1. Introduction

Aquaculture has become one of the fastest-growing sectors of global food production [1]. However, the rapid expansion of farming scale and the widespread adoption of long-term intensive production practices have raised concerns about the genetic sustainability of cultured populations. In many aquaculture species, however, reduced genetic diversity has been highlighted as a key concern [2]. Therefore, maintaining sufficient genetic diversity is widely recognized as a fundamental prerequisite for effective selective breeding, population improvement, and the sustainability of aquaculture genetic resources.

*Macrobrachium rosenbergii* is one of the most economically important freshwater crustacean species globally [3]. Since its introduction into China in the 1970s, the industry has expanded rapidly, making China the world’s largest producer of this species. Aquaculture production is primarily concentrated in Jiangsu, Zhejiang, Shanghai, and Guangdong provinces, which together account for approximately 80% of the national output [4]. However, similar to many intensively cultured aquaculture species, *M. rosenbergii* has experienced progressive germplasm deterioration over recent decades, largely as a result of improper broodstock management and prolonged inbreeding within closed breeding populations. This deterioration is commonly manifested as reduced disease resistance, slower growth rates, and precocious sexual maturation, posing serious challenges to sustainable production [5,6,7]. Although the introduction of new germplasm resources has been attempted in recent years to enhance genetic diversity, these efforts have so far been insufficient to meet the demands of large-scale genetic improvement and breeding programs [8].

Accurate evaluation of genetic diversity and population structure relies on reliable and informative molecular tools capable of capturing variation across genomes. Microsatellites remain one of the most powerful tools for population-level analyses due to their co-dominant inheritance, high polymorphism, broad genomic distribution, and ease of genotyping [9]. In *M. rosenbergii*, microsatellite markers have been applied to reveal patterns of genetic diversity and differentiation among wild and cultured populations across different geographic regions [10,11]. These studies have provided valuable baseline information for understanding the genetic background of *M. rosenbergii*. In the present study, microsatellite-based genotyping was used to conduct a systematic assessment of genetic diversity and population structure across 11 populations sampled from major aquaculture regions in China and several Southeast Asian countries. The results aim to provide a robust molecular foundation for germplasm evaluation, parental selection, and the sustainable development of future breeding programs.

## 2. Materials and Methods

### 2.1. Sample Collection and DNA/RNA Extraction

A total of 300 *M. rosenbergii* individuals were collected, representing seven Chinese germplasms and four foreign germplasms (Table 1). Prior to sampling, all individuals were visually examined for external signs of disease, including abnormal behavior, visible lesions, or ectoparasites. Only apparently healthy individuals were selected for subsequent analyses. The sampled individuals had an average body weight of 44.45 ± 7.23 g and an average body length of 16.44 ± 1.14 cm.

For each individual, muscle tissue was dissected and preserved in 95% ethanol at −20 °C until DNA extraction. Genomic DNA was isolated using the Tissue DNA Extraction CZ Kit (ONREW, Foshan, China) following the manufacturer’s protocol. DNA integrity was assessed by 1.0% agarose gel electrophoresis, and concentration and purity (A260/A280) were measured with a NanoDrop^®^ 2000 spectrophotometer (Thermo Scientific, Waltham, MA, USA). DNA samples meeting quality standards were diluted to a working concentration of 50 ng/μL and stored at −20 °C for downstream analyses.

For transcriptome sequencing, ovarian tissues were collected from 30 randomly selected adult female individuals among the 300 sampled prawns. The ovarian tissues were pooled at the tissue level and divided into three biological replicates, each consisting of tissues from 10 individuals. Total RNA was extracted using the Eastep Super Total RNA Extraction Kit (Promega, Beijing, China), following the manufacturer’s protocol. RNA concentration and purity were assessed spectrophotometrically (OD_260_/OD_280_) using a Nanodrop 8000 spectrophotometer (Thermo Fisher Scientific, Waltham, MA, USA). RNA integrity was confirmed via 1.0% agarose gel electrophoresis. cDNA was synthesized from total RNA using the M-MLV Reverse Transcriptase Kit (Invitrogen, Waltham, MA, USA) according to the manufacturer’s instructions. Both RNA and cDNA samples were stored at −80 °C in an ultra-low-temperature freezer until further analysis.

### 2.2. Microsatellite Markers and Genotyping

Transcriptome sequencing was performed on ovarian tissue of *M. rosenbergii* using the Illumina HiSeq™ 4000 platform (Illumina Inc., San Diego, CA, USA). After quality control and de novo assembly of the raw reads, a total of 70,269 unigenes were obtained. These unigene sequences were subsequently screened for simple sequence repeats (SSRs) using MISA software v2.1 [12]. A total of 53 microsatellite loci were preliminarily identified, and primer pairs were designed using Primer Premier 6.0 (Primer Biosoft International, San Francisco, CA, USA). The 5′ end of each forward primer was fluorescently labeled with one of four dyes: FAM, HEX, TAMRA, or ROX. PCR amplification was performed on a Veriti 384-well thermal cycler (Applied Biosystems, Carlsbad, CA, USA). The 10 μL reaction mixture contained: 5 μL of 2× Taq PCR Master Mix (Genetech, Shanghai, China), 1 μL of template DNA (~20 ng), 0.5 μL of forward primer (10 pmol/μL), 0.5 μL of reverse primer (10 pmol/μL), and 3 μL of ddH_2_O. The amplification program was set to be pre-denaturation at 95 °C for 5 min; followed by 10 cycles of denaturation at 95 °C for 30 s, gradient annealing from 62 to 52 °C for 30 s, and extension at 72 °C for 30 s; then 25 cycles of denaturation at 95 °C for 30 s, annealing at 52 °C for 30 s, and extension at 72 °C for 30 s; with a final extension at 72 °C for 20 min. PCR products were stored at 4 °C prior to electrophoresis.

Fluorescent PCR products were diluted uniformly and checked on a 1.0% agarose gel for quality control. Capillary electrophoresis was subsequently performed on an ABI 3730xl DNA Analyzer (Applied Biosystems, USA). For each sample, a 10 μL loading mixture was prepared comprising 1 μL of fluorescent PCR product, 0.5 μL of GeneScan™ 500 LIZ (Applied Biosystems, USA), and 8.5 μL of Hi-Di™ Formamide (Applied Biosystems, USA). Allele sizing and genotype calling were conducted using GeneMarker software v2.6.4 (SoftGenetics, State College, PA, USA). Finally, GeneMarker v2.6.4 (SoftGenetics, LLC., State College, PA, USA) was used to analyze the results, from which 20 pairs of primers with good polymorphism were selected, and four groups of multiplex systems with stable amplification were constructed (Supplementary File S1 and Table 2). Finally, 300 sample templates were divided into four groups to perform multiple PCR experiments, and the allele number, peak map, and genotype of each sample were analyzed.

### 2.3. Statistical Analysis

Genetic diversity parameters at both the locus and population levels, including the number of observed alleles (*Na*), number of effective alleles (*Ne*), Shannon’s information index (*I*), polymorphism information content (*PIC*), observed heterozygosity (*Ho*), expected heterozygosity (*He*), overall inbreeding coefficient (*Fit*), and within-population inbreeding coefficient (*Fis*), were calculated using GenAlEx version 6.501 [13]. Based on allele frequency data, genetic distances among populations were estimated using PowerMarker v3.25 [14]. UPGMA (unweighted pair-group method with arithmetic mean) clustering was then performed, and a circular dendrogram was generated.

Population genetic structure was inferred using STRUCTURE version 2.3.4 [15]. The number of assumed genetic clusters (K) ranged from 1 to 20. For each K value, 20 independent runs were conducted with a burn-in of 10,000 steps followed by 100,000 Markov Chain Monte Carlo (MCMC) iterations. The optimal K was determined using the ΔK method, and the corresponding bar plot illustrating population stratification was constructed. Based on the inferred genetic structure, an Analysis of Molecular Variance (AMOVA) was performed in GenAlEx version 6.501 to partition genetic variation within and among populations and to assess statistical significance. Gene flow (*Nm*) between populations was estimated according to Wright’s formula: *Nm* = 0.25(1 − *Fst*)/*Fst* [16].

## 3. Results

### 3.1. Characteristics of Microsatellite Loci and Polymorphisms

A total of 20 polymorphic microsatellite markers were successfully isolated in this study, including 11 dinucleotide, five trinucleotide, two tetranucleotide, and two hexanucleotide repeat motifs (Table 2). These loci were used to evaluate the polymorphism levels of 11 *Macrobrachium rosenbergii* populations. All loci showed high polymorphism. Across the 300 individuals, 202 alleles (*Na*) were identified, with the number of alleles per locus ranging from 5 to 27 (mean = 10.1). The number of effective alleles (*Ne*) varied from 1.862 (LSZX017) to 7.913 (LSZX065), averaging 4.063 (Table 3), indicating moderate allelic variation across loci. Differences in allelic diversity were observed among populations, as detailed in Supplementary Table S1. Compared with the Chinese cultured populations, the TG-ZJ population exhibited higher allelic diversity.

### 3.2. Genetic Variation Within and Between Populations

Genetic polymorphism analysis based on 20 microsatellite loci across the 11 *M. rosenbergii* populations (Table 4 and Supplementary Table S1) revealed substantial variation in both *Na* and *Ne*. The TG-ZJ population exhibited the highest allelic richness (*Na* = 8.250) and effective allele number (*Ne* = 4.583), whereas the MD-ZJ population showed the lowest values (*Na* = 2.200; *Ne* = 1.776). Shannon’s information index (*I*) further indicated that MD-ZJ, MJ-GX, and SL-GX all displayed relatively low genetic diversity (*I* < 1.000), while the remaining populations demonstrated markedly higher levels, with TG-ZJ presenting the highest *I* value (1.553). For heterozygosity, expected heterozygosity (*He*) exceeded observed heterozygosity (*Ho*) in nine populations, suggesting potential heterozygote deficiency, whereas MD-ZJ and MJ-GX showed the opposite trend. Correspondingly, the fixed index (*F*) was negative in both MD-ZJ (−0.169) and MJ-GX (−0.001), indicating an excess of heterozygotes, while all other populations displayed positive *F* values ranging from 0.034 (HZCX-ZY) to 0.152 (TZHL-DH).

Analysis of inter-population genetic differentiation (Table 5) showed that the MD-ZJ population exhibited the highest pairwise *Fst* values relative to all other populations (0.158–0.289), indicating pronounced genetic divergence. In contrast, the smallest *Fst* was observed between YZJD-YY and HZCX-NT (0.017). Based on Nei’s genetic distance [17], the largest distance occurred between MD-ZJ and MJ-GX (0.519), whereas the smallest was detected between HZCX-NY and HZCX-ZY (0.050). AMOVA results (Table 6) revealed that most of the total genetic variation originated from within individuals (77%), while variation among populations and among individuals within populations accounted for 13% and 10%, respectively.

### 3.3. Population Differentiation Analysis

**Figure 1:**
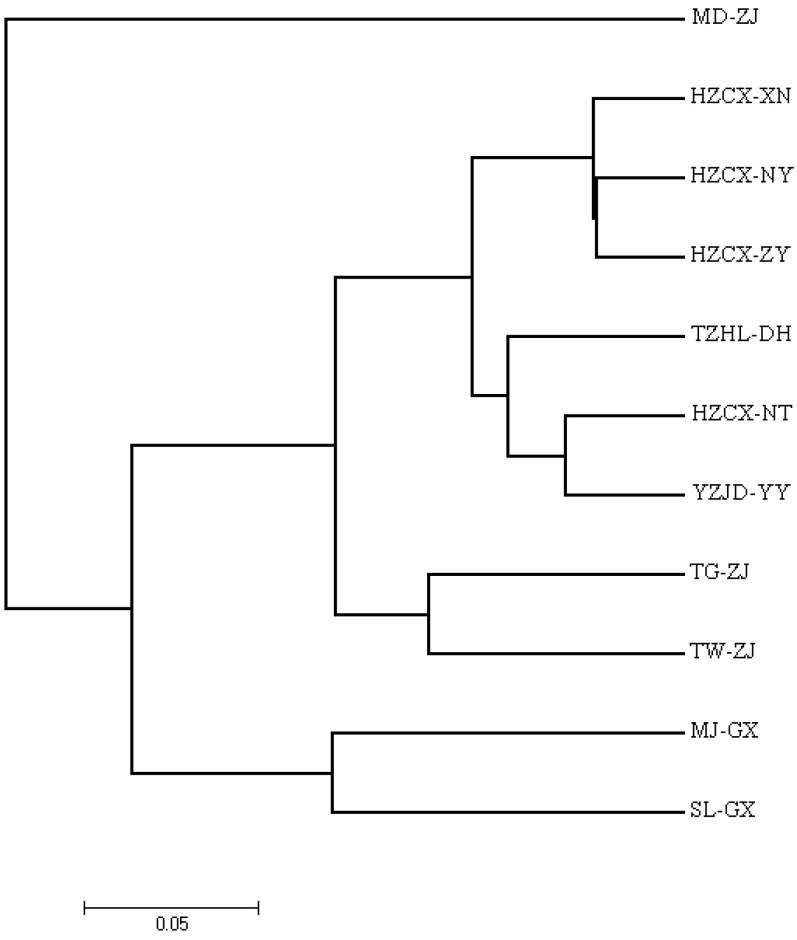
Representation of Nei’s (1983) UPGMA standard genetic distances among the 11 populations of *M. rosenbergii*, based on 1000 replicates [17].

**Figure 2:**
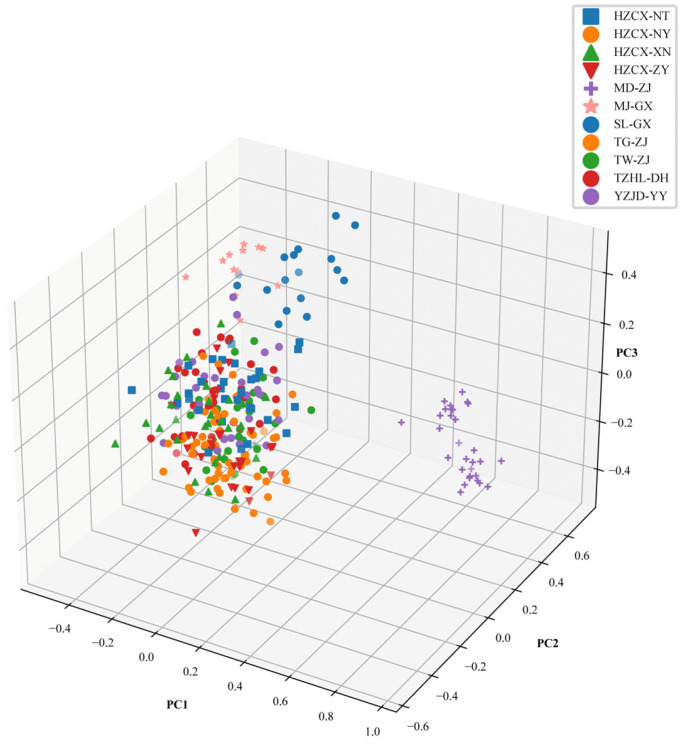
Principal coordinate analysis (PCoA) of 11 populations of *M. rosenbergii* based on microsatellite data.

The UPGMA phylogenetic tree constructed using Nei’s DA genetic distance (Figure 1) revealed that the 11 *M. rosenbergii* populations were distinctly divided into two major clades [17]. The MD-ZJ population formed an isolated branch, whereas the remaining ten populations clustered into a second major clade. Within this latter group, HZCX-NY and HZCX-ZY shared the closest genetic relationship. The results of the principal coordinate analysis (PCoA) were highly concordant with these findings (Figure 2). PC1, PC2, and PC3 accounted for 9.44%, 9.68%, and 3.90% of the total genetic variation, respectively. In the three-dimensional PCoA plot, the MD-ZJ population was clearly separated from all others, forming an independent cluster, while the remaining populations were tightly grouped, indicating strong genetic similarity among them.

**Figure 3:**
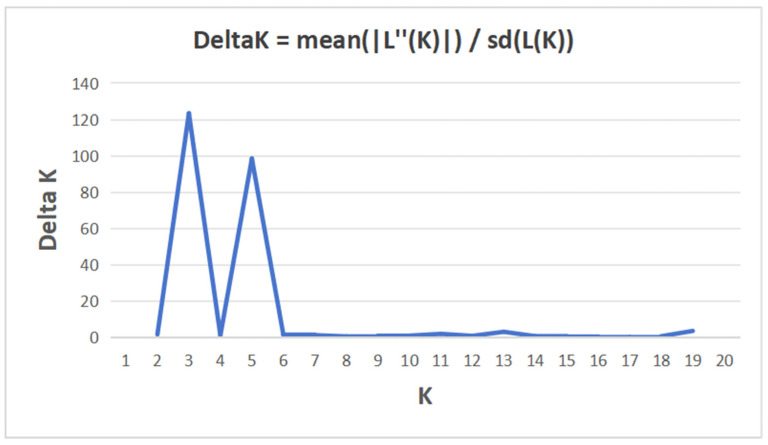
K-value diagram based on the ΔK method of the STRUCTURE analysis for 11 populations of *M. rosenbergii*.

**Figure 4:**
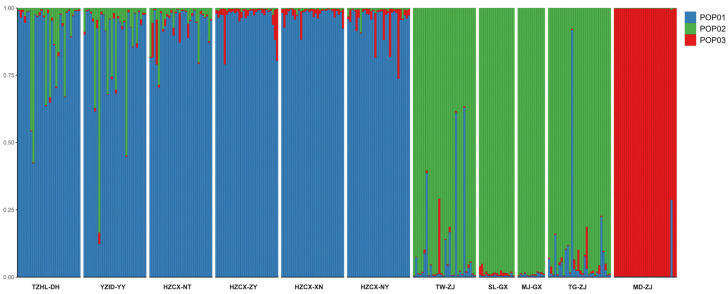
Bayesian clustering analysis of 11 populations of *M. rosenbergii* inferred using STRUCTURE at K = 3. Each vertical bar represents an individual, and colors indicate the estimated membership proportions in the three genetic clusters. Clusters of pop01 and pop02 were separate at a point of individual membership proportions reaching approximately 1:1.

Bayesian clustering analysis of all 300 individuals was conducted using STRUCTURE. The ΔK method identified K = 3 as the most likely number of genetic clusters (Figure 3), suggesting that the samples could be resolved into three genetic subgroups. At K = 3 (Figure 4), all individuals were clearly assigned to these groups. The first cluster comprised six populations (TZHL-DH, YZJD-YY, HZCX-NT, HZCX-ZY, HZCX-XN, and HZCX-NY). The second cluster contained four populations: TW-ZJ, SL-GX, MJ-GX, and TG-ZJ. The MD-ZJ population independently formed the third cluster.

## 4. Discussion

The microsatellite markers employed in this study generated consistent and comparable genetic diversity estimates across all examined populations. The marker set enabled the detection of population-level differences in allelic diversity and heterozygosity and supported the characterization of genetic structure among regional groups. These results demonstrate that the microsatellite-based genotyping approach applied here is applicable for population genetic analyses in *M*. *rosenbergii*, particularly in comparative assessments involving multiple cultured and geographically distinct populations. The genetic patterns revealed by these markers provide an empirical basis for evaluating germplasm status and informing broodstock management and conservation strategies in freshwater prawn aquaculture.

In this study, the overall genetic diversity of the 11 *M. rosenbergii* populations was relatively low and lower than values reported in earlier studies [18]. Notably, the MD-ZJ population exhibited the lowest diversity. As a descendant of the YG population described in our previous work [11], the MD-ZJ population showed a pronounced decline in genetic diversity compared with its ancestral stock, likely due to multiple generations of inbreeding within a closed population. Negative Fis values detected in the MD-ZJ and MJ-GX populations indicate an excess of heterozygotes relative to Hardy–Weinberg expectations. Such patterns may arise from several non-mutually exclusive biological and methodological processes, including heterozygote advantage, recent admixture among genetically differentiated lineages (inverse Wahlund effect), or genotyping artifacts such as null alleles [19,20,21,22,23]. Given the complex breeding histories and frequent germplasm introductions characteristic of cultured stocks, admixture-related effects represent a plausible explanation, although other mechanisms cannot be excluded. In addition, several domestically bred populations (e.g., TZHL-DH and HZCX-NT) displayed lower diversity than the Thai TG-ZJ population. This pattern further supports the widely recognized trend that intensive aquaculture practices often lead to reduced genetic diversity a conclusion consistent with a previous report [8]. Such reductions are generally driven by multi-generational artificial selection and limited effective population sizes, which can accelerate allele loss and genetic drift, ultimately eroding the genetic foundation of cultured stocks [24]. Reliance on a narrow genetic base for selection is a major contributor to declining diversity and may further compromise population adaptability and long-term viability [25]. It should be noted that sample sizes varied among populations, which may influence estimates of allelic richness. However, locus- and population-level patterns of genetic diversity remained consistent across analyses, suggesting that the overall conclusions regarding population structure and relative genetic diversity are robust. Therefore, introducing foreign germplasm—such as the TG-ZJ population—to incorporate novel alleles is of substantial importance for broadening the genetic foundation of *M. rosenbergii* breeding programs in China.

In this study, we systematically characterized the genetic divergence between Chinese cultured stocks and Southeast Asian populations of *M. rosenbergii*, offering important theoretical guidance for population improvement and intra-specific hybridization. AMOVA results showed that 13% of the total genetic variation was attributable to differences among populations, while 10% was attributable to differences among individuals within populations, indicating that the sampled groups may derive from distinct genetic origins. This inference was supported by multiple lines of evidence. Concordant genetic differentiation patterns inferred from multiple analytical approaches indicated pronounced population structuring among the examined stocks. Phylogenetic clustering based on UPGMA, Bayesian assignment using STRUCTURE, and ordination analysis via PCoA consistently supported strong genetic separation between the Myanmar-derived population (MD-ZJ) and all Chinese cultured populations, whereas the remaining populations were further subdivided into two major genetic groups. The concordance among these methods provides robust evidence for the existence of multiple genetic lineages within cultured stocks and strongly suggests that Chinese cultured populations did not originate from the Myanmar lineage. Overall, these patterns reflected distinct historical sources and breeding histories shaping the current genetic structure of cultured populations.

Genetic differentiation among populations was further evaluated using *Fst*, with interpretation based on Wright’s classification criteria [26]. According to this framework, pairwise *Fst* values between the Myanmar-derived population (MD-ZJ) and all other populations (0.15–0.25) indicate substantial genetic differentiation. Moderate differentiation (0.05–0.15) was observed for the MJ-GX and SL-GX populations from Bangladesh and Sri Lanka relative to other groups, whereas *Fst* values among domestic Chinese populations were generally below 0.05, suggesting limited genetic divergence and a largely shared germplasm background. This finding is consistent with previous inferences suggesting that some Chinese farmed populations may have originated from wild broodstocks in the Mekong River or Vietnam’s Dong Nai River [27]. Our results provide empirical support for the overall genetic homogeneity of Chinese cultured stocks. Nevertheless, given the ecological complexity of Southeast Asian river systems and the likelihood of anthropogenic gene flow across drainage boundaries, geographical isolation alone cannot fully explain the observed population structure [28,29]. Therefore, future research should prioritize the collection of geographically representative wild populations across Southeast Asia and implement comprehensive phylogeographic analyses to clarify the evolutionary origins and historical dispersal patterns of *M. rosenbergii*.

## 5. Conclusions

Maintaining genetic diversity is a central challenge for the sustainable development of modern aquaculture. Newly developed microsatellite markers provided molecular tools for germplasm evaluation and broodstock management in *M*. *rosenbergii*. The observed genetic structuring underscores the necessity for mitigating genetic deterioration in Chinese cultured stocks.

## Figures and Tables

**Table 1:** Location of populations and number of samples in each population of *M. rosenbergii*.

Sample Name	Number of Individuals	Source	Longitude and Latitude
TZHL-DH	30	Taizhou city, Jiangsu Province of China	119°55′31.90″ E, 32°29′48.23″ N
YZJD-YY	30	Yangzhou city, Jiangsu Province of China	119°34′35.76″ E, 32°26′25.06″ N
HZCX-NT	30	Huzhou city, Zhejiang Province of China	119°55′2.99″ E, 31°1′57.29″ N
HZCX-ZY	30	Huzhou city, Zhejiang Province of China	119°55′2.99″ E, 31°1′57.29″ N
HZCX-XN	30	Huzhou city, Zhejiang Province of China	119°55′2.99″ E, 31°1′57.29″ N
HZCX-NY	30	Huzhou city, Zhejiang Province of China	119°55′2.99″ E, 31°1′57.29″ N
TW-ZJ	30	Taiwan Province of China	120°11′44.13″ E, 22°59′39.37″ N
SL-GX	17	Kandy city of Sri Lanka	80°38′4.72″ E, 7°17′31.41″ N
MJ-GX	13	Khulna city of Bangladesh	89°32′25.18″ E, 22°50′44.31″ N
MD-ZJ	30	Yangon city of Myanmar	96°9′53.43″ E, 16°49′15.28″ N
TG-ZJ	30	Ayutthaya province of Thailand	100°35′15.59″ E, 14°22′9.24″ N

Note: Population codes are used consistently throughout the manuscript. Geographic coordinates are given in degrees (°), minutes (′), and seconds (″).

**Table 2:** Primer sequences and annealing temperatures for microsatellite marker amplification.

Locus Name	Abbr.	Primer Sequence 5′–3′	Label	Tm (°C)	Size (bp)	Repeat Motif
Unigene0011125	LSZX010	F: ACCAAACAGAGATGTATTGT	HEX	60	216–225	(AG)7
		R: TGTCACACTCATCTATACCA				
Unigene0011197	LSZX014	F: GATGTGGTTAAACAAATCAT	TAMRA	60	130–143	(AG)12
		R: ATAAAAACATAACCTGGGTC				
Unigene0011256	LSZX017	F: TCTTTACCCTATGTGATTTT	TAMRA	60	241–246	(GT)6
		R: GAAGAATCTCTTGTTTAGGT				
Unigene0011215	LSZX018	F: GAATGTAACAAGCATGACTA	FAM	60	227–256	(GAGCCA)4
		R: GGCTTTGTGCCACCTACAGC				
Unigene0011289	LSZX021	F: TTGGAACAATGATACCTAGT	TAMRA	60	290–308	(AC)10
		R: AATCTCAAAGAGAGGGCTGA				
Unigene0011343	LSZX023	F: TTGTAAATATCCATTCATCA	ROX	60	187–201	(CA)9
		R: TCACTGATGCTGGATGTTGC				
Unigene0011463	LSZX029	F: TGCCGACTAAAAAAGATTAA	ROX	59	291–301	(GA)11
		R: TGGTGCACTGTAGGCATCTA				
Unigene0011482	LSZX031	F: TGAGCTCAATCTCTCTTCAG	ROX	60	259–264	(AG)9
		R: CCAGGATCCTTACGTTTAGC				
Unigene0011500	LSZX032	F: CGGCTGCAAGGATGAAGTGG	FAM	60	197–203	(GA)11
		R: CCTGTGGCAGAGTTTCCCAA				
Unigene0011612	LSZX035	F: TGTGAGAAATTACTCGAGGC	ROX	60	244–261	(AG)13
		R: CTGTTAAAGACATCAGAAAT				
Unigene0011716	LSZX040	F: AGGACTGTTGGTGTATGAAG	HEX	60	278–295	(GCTCCG)4
		R: CACTCCAAGGTTCTCCCAGC				
Unigene0011814	LSZX051	F: CAGTGGTGATGATGGTGGGG	FAM	60	203–213	(GCAA)5
		R: GCCTGAAAAACTGTTTGGAA				
Unigene0011137	LSZX052	F: AATCTTCCTTCAAGTTTTCT	FAM	60	269–280	(GGA)6
		R: CTCGTCCTTTCTTGTGGGGA				
Unigene0011821	LSZX053	F: CCACTCACAGGTTTCAACAG	TAMRA	60	216–227	(GA)11
		R: TGATTTACACAACATTGGCT				
Unigene0011367	LSZX064	F: AAGCGACACCCGCTCGGTGA	ROX	60	222–237	(GAA)9
		R: CAGCCAGTTCGGGCAACGTG				
Unigene0011368	LSZX065	F: GTAGAAGCCAAAGAAGGTCC	TAMRA	60	210–236	(TCT)17
		R: AACCCTCGTGATGATGGCTG				
Unigene0011994	LSZX083	F: GTACCTGTAACGTACCTGCG	HEX	60	232–242	(CTC)7
		R: GCCGCGTACTTTTATCACAA				
Unigene0012007	LSZX085	F: ACTGCTACTACTGTTACTGC	HEX	58	268–282	(TAG)6
		R: TCACCTCTTCCCAACTATAC				
Unigene0012050	LSZX089	F: AATAGGCATATTATTACCCT	FAM	60	260–280	(CT)10
		R: CTGTAGCCATATGAATTTTC				
Unigene0011495	LSZX099	F: TTGAACCATAAGGATATATA	HEX	60	180–217	(AATC)5
		R: AATTACCAGTGCTAAATTTA				

Note: TAMRA, tetramethylrhodamine; FAM, 6-carboxyfluorescein; HEX, hexachloro-fluorescein; ROX, X-Rhodamine. FAM, HEX, TAMRA, and ROX indicate fluorescent dyes labeled at the 5′ end of the forward primers. Tm represents the annealing temperatures used for PCR amplification.

**Table 3:** Genetic variability at 20 microsatellite loci in 11 populations of *M. rosenbergii*.

Locus	*Na* (Observed Alleles)	*Ne* (Effective Alleles)
LSZX021	9	4.145
LSZX099	9	2.928
LSZX051	12	6.957
LSZX085	10	3.27
LSZX014	12	4.353
LSZX065	27	7.913
LSZX023	6	2.938
LSZX031	9	3.45
LSZX032	7	2.086
LSZX052	9	2.751
LSZX010	7	3.273
LSZX053	13	4.623
LSZX064	11	5.858
LSZX029	7	4.569
LSZX018	6	3.317
LSZX083	8	3.054
LSZX017	5	1.862
LSZX035	19	4.893
LSZX089	11	5.405
LSZX040	5	3.614
Mean	10.100	4.063
STDEV	5.160	1.556

**Table 4:** Genetic variability in 11 populations of *M. rosenbergii*.

Pop	*Na*	*Ne*	*I*	*Ho*	*He*	*F*	*PIC*
HZCX-NT	5.850	3.853	1.441	0.613	0.701	0.124	0.664
HZCX-NY	5.150	3.177	1.265	0.583	0.643	0.115	0.605
HZCX-XN	4.900	2.885	1.194	0.573	0.618	0.092	0.569
HZCX-ZY	4.500	2.970	1.174	0.595	0.621	0.034	0.575
MD-ZJ	2.200	1.776	0.554	0.432	0.360	−0.169	0.295
MJ-GX	3.700	2.283	0.930	0.512	0.507	−0.001	0.469
SL-GX	3.500	2.486	0.971	0.514	0.545	0.079	0.503
TG-ZJ	8.250	4.583	1.553	0.597	0.698	0.122	0.670
TW-ZJ	6.300	3.843	1.425	0.596	0.687	0.111	0.652
TZHL-DH	5.700	3.266	1.318	0.552	0.655	0.152	0.620
YZJD-YY	6.100	3.750	1.462	0.645	0.702	0.068	0.681
Mean	5.105	3.170	1.208	0.565	0.612	0.066	0.573

Note: *Na*, observed alleles; *Ne*, effective alleles; *I*, Shannon index; *Ho*, observed heterozygosity; *He*, expected heterozygosity; *F*, fixed index; *PIC*, polymorphic information index.

**Table 5:** Matrix of pair-wise *Fst* values (below diagonal) and Nei’s DA genetic distances (above diagonal) among 11 populations of *M. rosenbergii* [17].

	HZCX-NT	HZCX-NY	HZCX-XN	HZCX-ZY	MD-ZJ	MJ-GX	SL-GX	TG-ZJ	TW-ZJ	TZHL-DH	YZJD-YY
HZCX-NT	-	0.089	0.108	0.124	0.336	0.291	0.294	0.183	0.170	0.096	0.067
HZCX-NY	0.027	-	0.051	0.050	0.290	0.322	0.369	0.187	0.211	0.127	0.101
HZCX-XN	0.030	0.019	-	0.052	0.312	0.334	0.366	0.229	0.241	0.138	0.123
HZCX-ZY	0.037	0.023	0.021	-	0.298	0.358	0.367	0.230	0.248	0.149	0.131
MD-ZJ	0.159	0.158	0.180	0.164	-	0.519	0.475	0.431	0.461	0.394	0.371
MJ-GX	0.095	0.124	0.128	0.140	0.289	-	0.202	0.302	0.289	0.303	0.297
SL-GX	0.094	0.138	0.137	0.135	0.226	0.083	-	0.327	0.268	0.304	0.277
TG-ZJ	0.048	0.052	0.067	0.064	0.196	0.103	0.101	-	0.146	0.193	0.173
TW-ZJ	0.046	0.064	0.072	0.073	0.200	0.099	0.083	0.033	-	0.177	0.157
TZHL-DH	0.027	0.042	0.047	0.056	0.195	0.110	0.108	0.054	0.051	-	0.105
YZJD-YY	0.017	0.032	0.034	0.035	0.171	0.096	0.087	0.045	0.041	0.031	-

**Table 6:** Analysis of molecular variance (AMOVA) results for 11 populations of *M. rosenbergii*.

Source	df	SS	MS	Est. Var.	%
Among Populations	10	586.799	58.680	0.949	13%
Among Individual	289	2066.555	7.151	0.755	10%
Within Individual	300	1692.000	5.640	5.640	77%
Total	599	4345.353		7.344	100%

Note: Source, source of variation; df, degrees of freedom; SS, total variance; MS, mean square error; Est. Var., estimated difference value; %, percentage of variation. An intra-individual difference refers to the genetic difference caused by heterozygous alleles, and the size is related to the number of heterozygous loci in the individual, that is, the genetic diversity of the individual.

## Data Availability

The data presented in this study are available on request from the corresponding author.

## Supplementary Materials

The following supporting information can be downloaded at: https://www.mdpi.com/article/10.3390/ani16020270/s1, Supplementary File S1: Sequences information of microsatellite markers in Macrobrachium rosenbergii transcriptome data; Table S1: Genetic variability at 20 microsatellite loci in 11 populations of *M. rosenbergii*.
