# Supplementary material for: Genetic Diversity Analysis of 11 Macrobrachium rosenbergii Germplasms Based on Microsatellite Markers
Source: Animals (Basel). 2026 Jan 15;16(2):270. doi: 10.3390/ani16020270 (PMC12837990; doi:10.3390/ani16020270)
Supplement: Supplementary file 1 [file animals-16-00270-s001.zip › Supplementary File S1.pdf]

Supplementary File S1. Sequences information of microsatellite markers in *Macrobrachium rosenbergii* transcriptome data.

Unigene0011125
GCGCTGTCTACCTACGAAGTACACCTGACAGAAAGGAGGTAGCAGCAGCAGTAGTTA
GTAGTTACCGACGACAACCCCTAGTCGTAGGTTGACTCCGTTTCACCAGTCGACCGTC
AGTCCAGACACCTGCGAGATGCGGTAGCGTTAACTTGGCTTGGCCACAAACCTTAGTC
CGACTCCAGTCACCATTGAAAGCTCAAATCATCTTTTTATTTATTTATTTTTTTTTTTGGT
GTTGCATTTCTCGCCAATAGAAAGGCATGTCCGTTACAATGTTCTTCATGGAATCGAGT
GATCTTTAGTGTGAGGCCACAAGCGCAGACGAACCCTGAGCAGGCGAAGGAACAACA
GTTTTTTGAATCTTTGGGTAACATTGATACCACTCGGAAAATCGTCTCGGGTTTAAAAG
CACGCAACCACGTGAATATGATTCAGCAATCGAGATCGCTACCGCTGAAAGACTGAAC
GGAGAGAGGAAACCACCCCCACCAGCAGCAGCAAGGCTCGATCAAAGAGAGAGAGA
GAGAAAGAGAGTTTACAAAAATCTCAGCCTTATTTGCTACGTAAAGGTTTTTGTTGGTG
TTCAAGAGAATTAGCAAGTGCCTTCAAAGATGCATTTAACTGCTTTAGAGATGGAAGG
CGTAGTCGCTCCAGAGGCCAGAGGAAAGGACGAGGAAGAAGGGAATCCCGATGGAA
AGACCGACGTCGCGAAGTCTCGAGAAGCTCTGGTGGAGAGCGACTCGAGGAACAAG
CTTCTTTACACAGTGGAGGACAACCCACCTTGGTACACCTGTGTCGTCTTGGGCCTCC
AGCCCCTTTGGTGGGCTTGGTCCATATGAGTAGGGTTCATCTTCTGAATAATAATAATAA
TAATAATAATAATAATAATAATAATAATAATAATAATAATAATAATAATAATAATAATTC
TGCCAATGGAAGTCTCTTTCTTTTCATCTGGAGTGTGATTTTTGTATGGGAGTCAGTTCG
ATGGAGAGCTTTGGCTGCAAATTGTGTCAGTTGGGAGAGTTTTTTTTTGTTTACGCAGA
ACACATCAGTTTTTTAGGCCCAGAAGATATGTCAGTTTCAGTGTTTTTTTTTCCCAGTAG
AACTCACAAGAGTAGAGGTTTATTATTCTCTTGAAAAATCTGGATATAAAGATGAATAC
TGCGTCAAGAATAAGACAAAAGATCAAGATACAGAGTGGGCTAAATCCTGACAAAAA
AAAAACAGTCACCCTGATCGGGTTCTCGTTGTTTCCCGTCGCAGCTGATAAGGCCTCG
TCTCATTGGGGGATAAGCGCTCTGACGATCGCTCTACTCGTGATCTTCTCTCAGTACCT
GGCTGAGGTCAGCCTACCATTCTTCTCTTGGTCGAGAGCAAGGGGTTTTTCAAGGACG
AACCTTTACGTCTTCAAATTGTTCCCCGTTCTCTTGGCCGTGGTCCTCGCTTGGGCGAC
CTGTTGGGCGCTGACGGTCGCTGACGTCCTGCCTTCTGACAATCACGCGAGAACTGAC
CTTCGTCTGGAAATAATCAGTGATTCACCGTGGTTCCGCGTCCCCTATCCATGTCAATG
GGGTCTGCCAACAGTCAGCGTCGCAGGTGTCATGGGCATGATGGCAGGCGTCCTTGCC
AGCATCGTCGAGAGCATTGGGGATTACTTCGCCTGCGCCCGCTTAGCCGGAGCCCCAG
CGCCCCCCAAACACGCAGTGAACAGGGGTATCTGGATGGAGGGTCTTGGCACCGTTCT
AGCAGGCCTCTGGGGAACAGGCAGCGGCACCACCTCCTATTCCCAGAACGTGGGTGC
CATCGGCGTCACAAAGGTAGGAAGTCGACGGGTGGTCCAGTACAGCGCCATGATTATG
CTCCTCTGCGGCGTCGTAGGGAAAGTCGGTGCCCTCTTCATCACGATCCCGGAGCCCA
TCATCGGCGGCATCTTCTGCGTCGTCTTCGCCATGATCACGTCCGTGGGTCTGTCCACA

CTCCAGTACGTCGACCTCAACTCCTCCAGGAACCTCTTTGTCCTGGGATTCTCGCTCTT
CATCGGGATGGCTCTTCCAATGTGGATGCAAAAACCAGGCAACAAGGACATCATCAAC
ACCACCATACCTGTTCTGGACCAAGTTCTGTCGGTGCTTCTGCGCACTTCGATGTTCGT
TGGCGGTATTCTGGGTCTACTCCTGGACAACACGATTCCAGGAACGGACGAGGAGAG
GGGCATGGTCGAGTGGAAGGCTCAGCTGAAACCCAGCAGGGAGGAAGAGGGCAGAA
AAAAATTCTCCGTGACTTACAGAAATAAGTATACGACACTGTGACAAACATACACATAC
ACACATACAGCGCTACAACATAAATCTCAATATACAGATATATTTTGACCAGCTTACCAC
ATTCATCAAGAAAACATAATATTCACATCCGTAAAATATTCCCCCTCCCCTCCCATAAAT
TGAAATGCCCACAGTCTTTATGTAAACACCTCATCCCTTCTTGAGTTCTGTGAGATGATT
TCTGCTGTGAGGGCAGTTGACAATGGTAAAGTAGTCCTGCATTAAGAAAGAATTATGT
GTTTCTCTCGTCCGGTGAAAGAAACCAAGGATATGTTAAGAATGCTTCAGCTTTAATTA
AGCTCTGTCATCGGTACTGCTTTCTGGAAACCGAACCTAACTGCTGTGTGTACAGAGA
AGTGAGTGGTCTTTGGCAGGTCCACAAGTCCTCACCAGTTGCGCAGGCCTACACGTAA
GAGTCACAACAAGTGGCAGGTCCATACACGCAAGAATCAGCGCAAGTAACTAGAAGT
TCTGTACACAAGAGATGCTCCAGTAGTAGAAGGTCCACAAACAAAACCCACTGCCAGT
ATTTGACGGTTAAGCAAAGCTATTCGTCATCTGAATTCAAGCTCCGTTTTGTCTTCCATT
GTCCGAGGTCTTTTGATGCTTCTCCTGAAGCAGGTTGGAGGGAGAACTTCCTTTTCCAT
TTTTTGGTGCTGTTTGTGCATAAGAGAGGACCTTCCTTTTGTGTTTTAGTGAGAAAATG
AAGAAACAATCAAGTTATATTATAGTAGCGAGCGTTCCAGCTGACAATATTTAATTTGCC
AACGATAAATAGGCTACTGCAGTTGCTGCCTTCCAGTCAAGGACTGAACCATCTTCCA
GTCCTGCTCGCCAAATCCAGCACAAGTCCAGCGCAAGTTAGGCAGGTCCATACGTTAG
AATCACAAGCTGCGCAGGCTCACGGGCAAGAATCACAAGTTCAACAGGTCAGCAAAG
CAAGAGGCATCACCTATTGCGGAGGTCTACGAGCAAGAATCGCCACCAGCTGCGCAG
GCCTACACACAAGAATCACAAGTGCAAAAGGTCAACAAGCAAGAGTCATCCCCCATTT
TATTTAGACCAGTTATCCACAATGAAATTAGCACAATTATTATTTCTCAAGGTAACTTGC
ACACTTTAGATCAAAGCACATTTATTGGACCGGTAAGCAAAATGAATTATGTATACTTAT
TTTGTGTGTATTCATGCATTCATGATTACATCGTGGTATTAGACAAATAGTCACTAGTCA
AACTAAAACTGTATGCGTTATTTGTAAGTGTCTTATGTGTTTATGTTATTACCAAACAGA
GATGTATTGTGTATTTGACGTTTTCAAGGACAACCACTAATATTCGTGACCATAAGTACC
AGATGTAACACGAAAATCTAATCAGAAACAGAAATAACACCTTTGCAGAAATTTTAGAT
AAGCAGTTATGCTCAGTTTTAATGTTCAATCATTTTATCCAGACAAAAAACTGCCCCTA
CAATTTTAGTTTAGTGTTGTCTTCAGTGTCTGGTGATAAGATCAGCTAAAATAATATTGC
ATTATGGAAAAACATTATACTTTTGTACCCAGAGAAACTTAAAATAAGAATTGTTATTTC
AGATTTTTAGATGCAAATTAAGCTGACAAAGTTGCTGTCATGTATGGTATAACAGCATC
AGCTTTCCCCAATTGAATTTTGCCACTTAGACTGCATATTTGTCACACTCATCTATACCA
CAAATGTAAACACTGGGATTTTTATTATTTTAATTCTATATTCACTTCATAAAACGAGGGC
TTCCAACTTCACGACTCAGTGGCCTGATTAAACTACTCATTAATGATAGTTTCAAAAAC
ACAGTGACCTTTTCCCTTTGTTAGTATTGTGGTGAAAGCTATTCAGTACAAAATTCATAC
TCTCGACTGGCATAATTGTCCTCCAATTGTTCTTCATCCTTTTTTTCAATCTCTGGTCAGT
TGAATTACTATAGAATGTCTGCTTCATTTGGGCTTTCTGACCAGTTTTCTCACTGTTTTT
AACAGGTTACCACCTCCTCCTCTTGATTTGTCCCTTCCCCACTACAGGCTACTGCTCTTT
GAGTCCACAGCAGGCTATCATATACACTTTTCTCTTTGTGTTCCCATGGGATCACACTGC
TTTGGCAAGTTCAACCTGAGCTTTAATACTAAATTATTAAGGTCAGAATCTCAGTTTTAC
TGAACCAGTTGTCTTGTGTTTTTGTTAAGATATTCTCTAAGGCCCACCACTGGTCTGAC

AATTTCCCGATGTTCAGGTATCTATTTCTCTCCTTCAAAGCAAAGATTTAATTGAGAAA
ATTTCTGTTTTTTTTTTTAAGTAAAAGTTCCTCGTGTCATTCAGAAAGTGTTCAAGAGA
GACAGTAAAATAGTGTTAACTTTATACCTCCAAGATAAAAGTACAACAGCTGATTCTCA
TCACGAGACGAAGATTCTGTGCACAAACAGGACTTATTCCGAGACGAGATATGACTGA
AATCTTGCGGATGTGTTTCGTAAGAGATTCTCTAATTTAGAAAGGTTTAAGATTCTGTGA
TACTTTCCTATCAACTAGAACAAGAACAAAAAAACAACGGCACCAACGAATTAACAGT
GATTTTATTCCACGTGACATGTAGACTGTATGTATGGGTGCATGTATACATATATGTATTT
AAATTTGTACTTCATTTATTGGCTATCATCATTTCCTGGGTTTTGGAATTGTTAGGCCTAC
TTTGTGGGCAAAAATTCTTTTTAATTACCCTCCTACATCTTTTCAACAGTCAGTCCTATC
TTATCAACATGCTGTATTCTGTCAAGTAGATTTCAATGCACACCCACTTAAATACAGTAG
CCCCTTTTTTAGACCTCAAGATATGTGAAATTATAGAGTAGCTCAAAACTGTTTTTATTG
TTTGGTACAGACACACAAAACTGGTAAATGCATAAGCTGTATCTTTTTTTTAAGTTTATA
TGTAAATATTATGGACGTAAATTGTATGAAATTAATAAGCTTGACAAATACTGTGTATGT
GTATTCATACATACATAAT

Unigene0011197
TAGAATAGTACGATAAACCCGTGAATTTCACATTAAGTAGGACTGTTGATGGCATTAAAT
TTTGTTGATCTCAAAACTTTTGACAATGCTGTCCTTCACAAAGAAAAATAAAATTGGTT
TTTAGAATGTCGGATAGGGGATTGTAAACCTGTAGTGATAAGAGCAAGCAGAATTAGTT
GGTTATAAATGATGTGGTTAAACAAATCATTTATAACTAACTCACTATTCTAGTTAGCATT
ATTTTAAATACTATTGTACTATTTGACTCCTGCAAATTGTTACTGTAAGTATAAAAACATA
ACCTGGGTCAACAGTTCACACACACATTTTGTAGGTAGAGTATGATGCATCATCCTAAA
ATTTGATCAACATCTGTTTCAGAAAGTCACATGATGCATGAGTATAAATAATTTAGAAGC
ACAATAGAGCAGAGAGAGAGAGAGAGAGAGAGAGAGGGAATAGCTAACCAAATGCGAG
AGCTGATTTTTTTGCGAAATACTGCAGCCAAGAATATGACCACCATCGTAAAAAATAAA
GCAGCAAGTAAATCAAAGAGAGAGGGGAGTTGTACTGAGAAATCTGAAGGATGAGTA
CTTTCGAGTACCCATTCTTCTTAAGACTTATTATTAATTCACACCACCTGTGCATCTGATG
TCTAACCCCCGTCTCTTACGACGCTCCTGATTGGCCATTGATCGGCCAGTCACGGGGCT
GGAAGCTCGTCAGTCTCTTCCGGCCGTCGCCGAGTGGATATCTGCGCTGCGACCTCTC
AATTAAAGCTGCGGATGTTGTTAAAGGAATCAAAGCTGACAAATCAGAGGTCTTAAGC
ACTCAAGGAAGTAGATTAATTTTACATTATTCTTCTTGGGAAAAATTTGATTCTTTTGTG
CCTGTGCCTCGTGGAACTGACTAACAATGAGTACCAAGGTATCACTGTACTTTGACAG
GCATGGATCCTCGTGAAATACTGTATGTCTTTAAGTTGTAACTTTCATTACACCTAAAAT
TATTGTGAAAAACAAAAAGTAAAATGAAGTTACTTTACACAGTACACCTAATTATTGTT
TTCTTTATATACAATTATGTTTTAAAAGTAAAATACAAGCCCATCATTGCATAAAATGGCT
TACAGAATAACATTTTCTTGAGTGGTCTAGTATGAATGAACAGTTTTGCCCAAGGAGGG
GTAGTCTCTCAA

Unigene0011256
TTTTTTTTTTATGTATGTATATAGTAAATTCATATTATCTTTGCTTCTAAAAGGGAACTACC
ACATGATCCTGTTTTGTGCGTAGCTCTAGCAGGTTTTATGCGGTACAAGTCTCTTTACCC
TATGTGATTTTTGATTAAGAATAGTGAGAATGAATTTATCTCTTTCTTTAAAGACATTCCA
TTGACTTACAAGCTAGTTTTACATGTTTTATGCAGTTGTTTATAATTGCTTGTATAAAGTA
TTACTCTCTAGTCCATGTCAGTATTTTAATTCAGTTGTTTTGCATTCCTATGCATTGCTGG

TTGTACTGCTGTGAATAACAAATCCAGAGCTGGCATCACGTTTGTTTTCATAAGAAGCC
GTTCCTTTTGTATTTTTGCAGGCTTCTACCAATCACAGGAGTTTGTGAATTTTAGACTTG
TCCCATTTGAATTTTTGCTCATTTGGAATAAAATTTCTTGGAAGTTTTTCAGTCCCTTTT
GTTATCGACATGTCCTTTTTTTTTCCTTATATCTTCTGTTCGTCCGTCTCCGTGTTTTAGA
GTTTTTATGCATCTCAACCTCTTTCTTTCAAGAAACGTATCTGTAATCTCCCTATGAATTT
AAGTGTTTCCCTTCTGGATCAAAAGTGTATCTTCCGGTTATTAGCAAAATTGACAATTG
CTCATATCATTTCTTAAATTAGAATGTTTGTATATGTAAGGAGTTTGGGCTTCCTAGAATC
ATCATATGTTGGCACCGTACTGTCCACAAGTTACCAAGTGTTATTTGACCTCTGCAGTTC
ATTTCTGGTTTGGGCATCTGCTGTAGACAAGAATCTTCAGTTGCAGAGATATGAATGAT
GTTGTGTATTGTCTTCATCAAGCGATTGACTGTGCAATCAGGCCCCATTTTTTCACTTTG
TCTTCTCGTATTTTTCTTTGATGTCTATAGAGTTATTCGAGATAACCCGTTGCTTTTACCT
TTGCTCTTACATGAGAACCCAGCTGCCTTGTATTGGTACCTTTGATAGACCATATCCAGT
ACAGTGATTAGTAATACTTGCATACCTGCTGGTGAATGGTGTGCATTTTTAGGTTGGTGT
GATAACTCCTGGTGCATATGTTAATCAGGACATGATGCTCAGCTGCGCTGAGTAGAATAT
TTGTGCGGTTTGCTCACGGTCTCTGGAAGGAAGAATCTCTTGTTTAGGTTTTTTGCTGA
AGAATTTGGGAGTTAACGGAAGGAGAAGGTATCACAGTCATGAATCAAGATCTGTGGT
TCTGATTTTTCAAATTGGTTTGCATTAAGGCTTGTGTAAAACTATGGATGTTATGGCTTTT
AAAAATTAAGAGTTTGTCGTTCTTCATATCAAACTTGTATTCTTTATTTAAATAATGCATT
TTAGGATTAATTTGCTTGCTTCACATGATAAAAAAAAAATCCTTCTCCAGGTGTGTGTG
TGTGCACCATAAATATATGAAAGCACCTGAGAAGGATGCGTTTTCATCTCTCAGAATGT
AGTGTTTGTTATACCGTATGGGCATCACATACTATGAGTGATCATGTTACATTTTGAAAAT
AGTACATGTAAAAGTCAGCTGATTCATAAGTGAGCTATTACAGTAACCTTTCTTTCTGA
GGCGCTGGAGATTGCAGAGTTGTAACTGACACATCCTCATTATCTGTCATTCTCAGAGA
ACCTTGTACCATGGAGAAGTTCGTGGAAGGTAGATCATGTGGTCTTTTGTTCGCTTGAT
CATGAGCGTACGTTGTTGCCCCTATCGCAACTTGTCTGTTTCAAGTGACAGATCCATAG
CTTGGTTATGGTAAATTACCATCACCTGCCTTTATTTGCAAATCGCCCCTCACATTCATTA
AGGTTCTCATGGCAAGACAGTTTGTCTGAATGTGTTACCGCCTCATTCTCCGCAGTGCT
CAATGTGGAGAGTGACCAGAGTCCTTGTATACTAATGACTTGGGCCTCTGTTTTCAACT
CGTAATTCAGCTGTTCTTTCCACTGGTGATGTCTTATTGATCTTGAAAAGCTATGCCTTT
CTTTAGAAAGCAGAAATACTGATCTAGAAACACAGAAATCTCCCGGATGTAGGAATGT
AGTCTGATACTGTATTTTCTGCAAGAGGTGGTCAGGAAGATTTTCAGGTTAACACCAAC
CCCGACATTTTGTTACGTTTGTGCTACTGATGATGAGGCAGACCGCCTGCACCTATTGT
CATTGTATATTTTGTAGAATATCATGTATTTAAATGGTTATGTTATTCTAATACACTTAGCG
TCAGCACATTAACTTTTGACGTACAGTATATAACTTGCCGTACGTGAAGGGCATGGTAG
CTAGAGCTTCAAAGATTTTTTCTACATTGCAGCTTTTTGTTTTACAAATAAATGCATGTAT
ACACATATACTATATATTATGGCATATGTAATTTCAACCCTTCATATTTGTTGCAGCTGTTG
TTGTGCGTTACGATACATATTAAAAAGCTATAGTTTTTTTATCATCTGAATGTTAGGCAG
AATCATAAAGGGTAAAATGGGTGCCCATCAATGATGTTTAATGCTCATTCATGATATATTT
ACTTTATCTCTGTTATTCACATTGAGGGAACATTTCTCATAGCACCAGTTGACAAGAATT
ACTATCAGCAATATTAAAAGGTATGCCATTCATTTTTGGTTTGGGCAATCTTTTGTTTCG
ATGATTAGTGATGTTATAATTACTAACGCAGTAATGATATTGATCTACTTAATTACACTAAT
AACGTTATGTTTTTCGTTGCGATCGAGAACACTTACGTCAAAATAAAAGTTGAGGGTAC
CTTTTTCTCCGTTTTGTCACATCCCAGGCCAGTAGTGAATTTATGTTGGGAAAACATCA
AAGAAGGGTGTAACATTTTGCCAAGAGGTTTTACGCCGCTAAGGAAACAGCTATGCAT

ATCATGATGCTTACAAGTTTTTCATATTTTGCTTCTGATAAGTAATAAAATCAAAGGGTG
TGAAAAATTTGGATATTCATACGCAGATGTTATTGTACTGTATTTGTGACTGGCAAATATA
TTTGATATTTGTATTGTGTAAGATTCATGGGAAGGTATGAAAGTGCCTTGTGTCATTCTT
CTTCCGTAGTCTAATTATATTTGCTGTACATCACACAAATTAGGTACCATATCAAGAAGT
TTTGATTTCTCTCTTTGATATAATTCAGCGGTTAGGAATGATCGTGTATTGACTAATATTT
ATTTTCTTGTCAATAATAAATCTTTCTTGTGAGTATCTTGTAGGAGGTTTCAATTAAAGA
ACTGCGAAAGGCAGATACAGTGTACCACTGTACTGTAATAGAATGTCGAGATACATTTG
CCGGGTAAAGGAATGTGACAGTCATTCAGTGCATACTGATGGTAGATTGTTGTTAATAT
GTGTGAACTAATGTGTTGAATATTATTTTCAGCTGATGACACTGACCATTTCAATTGTAT
TTTTTTTTCAATTCCGCTAGAAAAAGACCGTTTCAACACGAATTGCGCTTCAGACGTTT
CTCTGTATATTAGAGAGTCGAAGCAGGCCCTTTCCCCTTGTCCCGAGAGAAAGGATGTT
GTTAACATACTAACCATGCGCTCTTTTGTCAAAGTGTACAGCTTCATTTATTTGCTACAG
TTTGTTTTATTTGTAGTTTGCTGACACTGACTTGATGTTCGGGTGTTTTTCCACTTTGTG
CAATTCTCCTTCACCTTCCAGTGACTGACAGGCCCTTTAGTAAGAAAGGGTGTCACCC
TTTGTTTAAAGACTAGTAGGGACTTGTGAAGGCAGTCAGTTCCAGCAACTAGGAAGAC
TGTTAGTATTGCACTGATTTCTGGTGAAAGTTAAGCTGCTGTTTAGGAAGAACACAGTG
GGATGTCGTAATCGAATTTGAGTCTCAAGGTAGTACAGTATTGTTTGTATGTTATTTATTC
ACGGTGATATTGGCAATATTTGTTTATTCCCCCTACGTATATCAAATCAAATTCACATTGG
CATGTTTTAAAAGACACCGATTTCAAATACTAGAGAGCCGTGATTGAATTGTTGTGCTC
ATGGAAATCATGTTTAATTAGAATAAACAAAGGATTTGCATCTTAAGTGGAGTAGACTC
AGATATTTATAGAACTATAATGGAAGTGTTTGTTACCTATTTTGCTACAAGTTTTGCATAA
CCATATTCATGATGTGGCCAGA

Unigene0011215
TGGTTAGAGGGCTTTTGGCTGATCATATGAGTGAATATGTTCGGCTTGTCTTTTAGTCAT
TGTGCAGCACCTGCACCATACCTGTGATTCCAATTAAACGACCTGTAAACCTAAAAGTG
TGGTCCGACTCCTGGCCATGTCCAAGTGGTGTGTGATAAACCAAAAAGTTGTCGAGGT
ATTGAAGTTGAATTTAAGGGTTACGGTAAAGTCCACTGGACAGAGCGCCATACCACTG
GTACTGGTGACAATAGAAGAACTGAAACCAGGCATTATACTAGCAACGAAGTCTACTAT
AAAAATAAATATTATGTATGGGGTAATGGCTCATCCACATCAGAACTACCACCAGGCAC
ACATGTGTTCAACTTTAGTTTCCTACTTCCACAAGGGATTCCATCCTCTTTTGAGTCTCA
TATTGGCAGAGTGAGGCATCATTGTAAGGCAAAAATGGATATTCCTTGGAAAGTGGCCT
TGGTAGTTTTCTTCCTTCAGGTCCAGGTTGTATTGTGGACAACAATGCCGACCACTATT
TCGGTTGTATTTCATAATCCTGCAGCAGTTTACTTCTCTGGTCAGACTATAGACAAGACA
TGCCTGCGACCTTACTCGGTCAATACTCTGTATGATCTCAACACTGATCCACAAGCAAT
GATGAAGATTGAAGTTTTGTAATATCATTAATATTGATTATGAATTTGAGTTTGAGGTAG
AGCCCTCTGGTTGTCATGGAGATCTTGAGCACAAGGTACCAATTGTAATTATGAATCCT
ACTGCATACAATCCAAATATGCCTCCTCCTGCATACCCAGGTCCACCCCAGCCATCTGC
TCCAATGTTTCCTGGTGCAGTGCAGTATCCAGGGATGCCTATACCTGTATACAACCCATG
CATGTTTGGAGTTGCAAATTTCACTGAAGATGATAGTGATGATGAGCATGGAAACAATG
GAATGAAATTTGCTCCTCATTACATATCTTACACTCTTGTTAATGCGAAGCACCAGCAGT
CAGGTTATGATAATTCCTCATTTGGGAGTGGTATGGTAACTGAACGAAAGCGACATGGC
AGTGGAAGCAGCAGTAGCAGGCTCAATTCCACTGAAGCAGTACTTTAACATCTTTGCC
AATGGCTTTCCTGGTCAACCCCCAGTAGCAGGAGCACTTGTACCAGGACAGCCTGCAC

CAGGTTTTGTGTCACCTACGGCACCAGGCTTTGTGCCACCTACGGCACCAGGCTTTGT
GCCACCCACAGCCCCAGGCTTTGTGCCACCTACAGCCCCAGGCTTTGTGCCACCTACA
GCACCAGGCTTTGGGCAGCCAGAATACCCAAGTCAACCGTCATATAATCCAGCCATGC
CACCTCAGCCAGGTTTTGGAGAAGTTCAGAGCATGCACACTGTGATCAAGGTGATAAA
ACACCGTTAGTTATCCATCACCATCACCATCATCATTCAGGCTCAGAGTCTGAGAAAGA
TGCACCTGTGTGCATTAATGAACCTGATCCAGTCCCAGAACCAGTAACTCATTCAGTTC
CTGAACATGATAGTCAGTCATCTCATAGCAGTAGTAGCAGTAGTAGTAGTGAAGAAGCA
GCAGGAGAAGATCCAGTGCCAGAAGAGGAACCAGAAGAAGAGCCTGAGGAGCCAGA
GCCAGAGCCAGAGCCAGAAGAACCAGAGGCAGAGCCAGAAGAGCCAGAGGAATAGA
AGGCCATATTTTCCCTTAGGTTTGTGGTGATGGTTATTATAGCATCGTTCATAATACTAAT
TGCATTTATAGTTATCTGTTTTACAGTGCCTTTAGTCCATTGTTATATCATTAAATGTATTT
GGCATCATGAAACCTTGTATTCCATCTGATTTCTTGAGTATGGCCTCTTAATACATAGTA
GTATTTTGGGTGAAGTGGTATTCCGTCCATCTTGACACTAAAGTTATCAGGAGAAATTA
ATTTAATGTTAATCACCTCATGGATTTGTATGCTCAGATTTTTCAAAATGAATTTTATACT
TGTATGACTAGCTTTCAGCCAGAATTTTAAAAAAGTGTTTTTAATTATCCATGTAGTCT
CTGTAACAAGCATGACTACATGTGCTGTTTGTGGTGTAGAAGTGGGCCACTATCACTGG
TCTTGAGAGTTGATCGTTCAGGGTTTGTTCCAGGTGAAAATATGAGCATCAACGCTGA
GATTTCTAATATGACACGAGTTAAGATTAACTTTTCAAAGGCACAAATCCACCAGAAAA
TAACATACTTTGCTCAAGGTAAACAAAAGGCGGAACATCGTAAAGTGGCTGAAAGGA
AGCACTCTGAGATTGCTGCAGGTGGGGATGATATATGGTCAGGTGATGCATTACTGATC
CCTCCTCTTCCTCCATCTCATCTTCACTGTAAATGTATATCTCCACTTCGTGCATCCTTGT
ATGTTCATTATTTTTTCCTAAAATGTTTACTTTACAGTATTATTAGTTTTGCAGCAACTGT
CCTGTTATTTATTTTCAGTGTGCACACAGAGCAAAAGATGGTTGTTAATATTACTCTTTG
TTCATATTGACAGTGTTGTAAAGACATTTTTATTTTTATTAAAACTGTTAAGTGTCATTAC
TTATAAGGAGAAAGGGCAAAATAACTTAAGTTTCAAAATTGATATTAAGTAAGGAGAGT
TAATGAGAAAATGTCATTTATAGGCAGGGGTCATACAGCATTCTTTTTGTTATGTTACCT
TATTTTTGCTTATCCTAAAATTAGTCGTGGTATATTAAGGGACTTGATCCTCACATTATGT
GGGTCATAACGTGTTAAAATTTAGCACCCACTAATTAGCAGTTTACAATTGATATATATTA
TTAGCAAAATGAAAGAATTGTAACTTTCACTGTTGCAAACCCTATTTTATTTTAAGTGCT
GAGAATAGGAAAGTATAATAAAAGATAAGGAAGCACCTAGTTTTCATTGGAGTATGCTG
AGAGTACTGTGTTCCCAATATTATTTGGTTTTTCATGTGATTATTTTATGGCTTATTTTCTT
TCCAACAAACAGTAACCAACACAGTAC

Unigene0011289
AAAAAAAAAAAATGCTGAGAATACAGTATGCACAAAATGATGAAACGAAACTACCTGT
ACATAATGAGGTTGAATTTTCAAATATTTTGGAACAATGATACCTAGTACTTTACTGTAT
AGGTTCTTACTATAAAAACTGTCACCTATTGTAATCTCATATTTTCTCATTTGTGAGAAA
ACATTAGTGCACTCATGTTGAATAACACAAAATATAATTTGACGATAAACTTGCTCAGC
CCTCTAAGTCTGTAAAATTTGGTGAAAAGCTAAAAAAAAAAAGGCATGTTATATAGTCA
TGATGAATGAGATTTGAAAATGACTCAAATTATATACCAAATGATAATCAGTCAGCCACT
GAACATTTCTTCCCAAAATTAGTCCAAAACATCCAATTATGAGGATAAGCTACCAGATC
TTTCATACAAGAGATCATCACTAGTCCCTTTTCGCTGGCTGAACAATGCCAAAAACTAA
AATACTCAGTGACTCTTAATCCCAATTAGCTATTATCTGTTAGCCATGGTAATTTTGTCTC
CTACTTACAGATGACCACCACAACTTTCAGTGAAAATTCATGAAAATAAGAAAAACTG

CCTGAAAACCCTTTAAAATCCTTATGATACAAAACTGGCAGTAGATTAAGGCTCCCATT
TTAGACAAGGATAAGTAGCCGTGGGGAAGTTATTTACGGAGCAGATATGGACATGCCA
GCTCAGAAAATCCTCATCAGATCCTTCAAACTTCCGCTTAGAAGCTCTGCGTCTCACTT
CTTTCAGACAGTTTTGTACTCTTGGATTTTCCAAAGACTCTTCCTTCCCATTTAACCAAA
ATTTGTCCTCAGTAAATGTTGGTGATATGGATACTGTTGTGGTGGCACACATCTGATCTT
GGCTAAGTGTGAAGCTAATGGAGTCATTCAGAGGTAAGATGAGTTTCTCATCGCGTTTT
CCCCAATATTTGATAACAGCAATATTGACAGGCGCAGTGCAGGTTACAGACCTCATGTT
GAAATCCTAAAGTTACTAGACCAGCTATCACCAAAATAATATTTAGGCTTTAAATTACAG
TCTACGCTACGGCAGATTTAAAGGCCTCGGACCACCACCGTAATTTGACTATACCAACT
GGTCTTTCGGTAACAATTCACAACTACTCCAATCTTAACAGTTCACTCTGTGGTTTAAA
CATACAAGCTTGCTCGAAAATGCAACACTTTAACAACAGCTAGTAAGATTTCAAATTCA
GACACACACACACACACACACACCATCCTCCCTAACATTGCATAGTAACATTATAGGAAAT
TTGTTTACACATATCACAGATACACTATTCAGGTATTTAGCTCCCTGAATCGAGAGGAGA
CAACTAAAAATTTCTGCATGTAATCTGAGAGCAGCTTTTTACACCGGGTCCACATCTTA
CTCCCGGTCGCCCATCCCTAGGCAGAATCGTTTATTGCCTTTGGCATTCCCGTCTCACA
GTCGAGCAAAGCGGCAGAATCGTCTAGTACGCAAGGACCGCCACCTACCTTTGTGTGT
ATGATAAACTTCAACTGTCCACCTTGACAATCCTCATCTAACAAAGACAGCAGCCCTTG
TGACAAGGGCATGGTTTCAGTCTGGTGGCCCCTGACATAATCATTCATATTTTGAGATG
AGCTTGGTGGAAATGCATGAAGCAGGAGGAAGACTGATTACCTGAGTTCTCGGCTTCG
AATCTACTCTGATATCAACAACAATAGCAAAATACTTTATCAAATACAGCACCTAACTTC
AGCCTGCCACAAAGTTTGTGTCTAGGTCTGCTGAGTACAAATACAATTCAATATCCAAA
AAATTTTAACATCAGTGATAGTTAGCTGCATGAATTCATCAATAACAATGGCAGGTACA
AATAAACAAGCGTTGGGGCCTGCGTCAAAAGTATAGCATACTTTCTGACTGAGACTAC
CCTGTGACTCCTTCACATCATTGTACTGATGAACAAGACTTGCAACTGAATGAGAGGT
GTCACTCATGTAAACACAAGGAGGGAATGTATCTTGGCAAACGGCATGCATCTCGTGTA
TCAACAAGAAACACCAGTGTAAATATTAAGGATCACTTAAAAAGAAAGTTTCAAGGGG
AACATATCCCCTTGACCGTGGGAACCCAACCTGTAACAAAAAAACAACCTGCTACTCA
AAATCTCAAAGAGAGGGCTGATCACTTCTGCCAACGAACAAGGCCTCCGTATATGCTG
CGACAGGCACTTCCCGAGCCTTGACGGGCCACAGAAGACAGATCCCCTTCGACTCCAT
AGACTTGTCCCAGAGCAAAGACCAAACAAGCATAACCAGCAGCTGATGATAAGATGG
AAAGTCAAACTACTGTACTGCTATTAGAAAGGGCAATCTTCAGGAAACTTCCAAGACT
GATGCTATCGGCCATCGTCAGGTCACAAAAACTGGACCAATCTTTCTCCTTGATAGCCT
CCATTATCGCATCTAAGCGTAATAACACAGATTCCTTTATCCGATGCTGCATCAAACGTG
AGGTCTGGACAGTTCTCTTCATGCCCTCTGTACTGCCTACTTTCTTACGGCCAGCATTTG
CTACACATATGATGACCCTTAGATCTGGAAAATGGTCTGCAGGTGCTATAGGACGAGCC
ACTGAATCAGTGCCATCTTCTAAATCTCCTTTGGCCAAACCAGCAACACACGACACTTA
TAGAGGCCTATATATTGGCTGACAGTGATATGGCAACGCTCCCCGGTGGCCTGTACAAC
ACTGGGGTCCACTCGTCTTAAAATCGCTATACAAAACGCATCCGCATCTATTTTTAACCA
CGACTTTAAATAGGAAATTGTTCCTCGCGGTTATTCACCCTTGGGGCGATTTGACAAGC
AC

Unigene0011343
TATAAATCTCTTAAAATCAAATGCTTAAAACAGCTGGATATAAACAGTTATGTGACAGCC
ATGATTGCTGTCAAGTGAAGAAAGTGACAGCCAACATATGTAAATCACTTATTATATCTG

TATACTGTATTGTCTAATGTTGTACACTGCAGTGCATGTTCTTCCCAGCTTACAATGCCA
GCAAAGTATATGCAGGTATATGAGAATCAGAAGGGCTAGAAAGAGTAGTTTGAAATGA
TGGATAAAAAATGAAGTATCACTTATGTCGAAGCATCACTGCTACTGAAATCTTTCTTTA
CTACTGGATGATACCCAAGGCAGAATGTCTTCACTGCTCCAGGATGCTGCCTGTAAGCA
AGCTTCTTCACTGCTGCTGGATGCTGCCTGAGCCACAGCTCTTTCACTGATGCTGAAA
GCTGGCTGAGCCAAAGCTACTACTCTGCTGTTGGACAACACCTAGCCCTAAGCTCTTT
CACTGCTGCTGGGCATTCCTAGAACCAAGCTCCACTCTGGAAAATGTTGGTAACGCTG
TTTACACTCACACAGAAACTGAAAAAGTCTTTGAGGGGTGGAGAAAACCTGGAGAAG
TTTCCAATGTTGGGGGGGGGCGTTGGATGGCTGGGTGCCATGTGAGAGGAAGGGCAGT
AAGGCTGTGTCAAGGAACTCACTAAAAGAAAGGGTTTTTTGGAAGGAGGGTGGAGCT
GGGGAGCAGTTTCCCAAGCTGGGAAGAGGTGAGGATACAGTGTTGGATGGGAAAAGA
GGGCTAGATAGACGTCTGACTTGCTAGAGCTTGTTTTGTTTTGTGTTTTTGTTTATGATT
TTTCCATATTTTACTAAAGTTTATGCACAGTTCTGTACATAAATATTTGTAAATATCCATTC
ATCAAATAATGCCATTAATTGAAAGTTTATGACAGCAGACAGGATAAGCAAAGCCAGTA
GCTCTTTGGTTTGCTTCATTCAACTGAAAGATTATAGTACATCCAGTATTATTACAGTAAT
CCCTCGAATATCCAGATCTTCATCATCCAGAACTCGAAGTTATCAGCATTCCCAGACCA
GGGACCAAGGACCAAGGATATAAGATAAATTGGCAATGCCGCCCGGCAAATTGAAGGA
AAGAGTGACATGTAACTTGCAATTAAAATACATCAACCACAAAGCAAAATTGCTACTG
ATAACAAAAAATTGGGTGCTACCTTAACCCAAGCTCCTTCACTGATGCTGGATGTTGCC
CTAGCCTAACCTCCTTTACTGCTCTTGGAAAGTCTAGGTTCGAACTCCCTCACAGCTGA
CGGGCATTGCAAGACCTGAGCTCTGCTCTAACTCCTGCTATGGTGCTCAATTCTTCCTG
AGCCAAGTTTTTTCATTGATGCTGGATGCCATATGAATTACTTGACTGCTGCTAATACCT
GCACAAGCTCCTTCACTACTGTTGGATTCAGCTTACAACCAAGCTCTTTCACACACAC
ACACACACACTAATCCTACTACAGTGCTGAACTCTACTTAAGCCCAAGCTCTTTCAATG
ATGGTGGATGCTTGCCTGATTCATACTGATGCTGAATGTTGCCAGATTAATTCTCTGATG
CCGGATGCTCCCCAAGTTCTTTCTCTGATGCTGGACACTGCCCAAGCATCTTGATGCTG
CTAAAAGCCACCCTTGTGTCAGCCTCTTCAATGCTGCTGAACTTTCATTGTTGCTGGAT
GATACCTGTGATAAAACATCTTCATTGCTGCTAGATGGTGCCATACACAGAACTCCTTC
ACTGTTCCAAAATGCTACTGAATGCTGCATAGACCACACACTTTCTCTGCTGCTGGTCA
TCAGCTAATCCTTAGCACCTTCACTGCTGCTGGATGTTTCCCAAGCCAGAGCTCCTTCA
CTGCTGGTGGGCGTTGCCCAAGCCTGAGCACCTTCATTGTCTCCTGAGCTAGATATCCT
TCACTGCTCCTGGACAACTCCCTAGTGTCAGTGCTCAAGTGCCTTTACTGCAGCTGGAT
ACCATAAGAGCTGAGCCAGAGCACCTTCACTGGTGCTGCTTGCCATTCGAACCAAGGC
TCCTTCACTGCTGGATACCACAGAAGGATGAGCTCTTTCACAGCTGCTGAATACTGCAT
ATGCCAAATTTTTCACAGATGAATGTTTTTATCTACTAAGTATATCACATTACTGTCCGAG
CTCATTGGAAGACTTGTGCCAGTGCAAAATTTTTTCTTACAGATAATGACTATTTTCAAC
TAATACAACTTGATAAATTATTATACATCTACACATAATCAGAAACTTTGGCAAAAGTTA
ACTCTATTTCTGTACATCATATTAAACCATAATCTTCTAGGATAATTCATGTATATGGGTTT
GATATGTGGGTAATCCATTCAAATACTTAAAAATTTCTAAAAATACAATACAAGCTACTG
GAAACTGCCCAAGCATTCCATTTAAAAATTCAACACTCTTATTTTATCCCTTACCAGTGT
TGAGCAAAGAAAAGGTTTATTTGGTGAGTAGACTTTTACAATGACTGTTCCTATTAAAT
CCCATATTCTTCAAAATAATTTTTTAAATCTGAGACAAATTTAGCTAGAGCTCATTCATT
GCTACTGGACATCATTTGAGATAAGGCTTGCTACAGTGCTACTGAACACTTTTCCCAGC
CAAGACCCTTCCATTGATGCTGGATATCACCAAAGCTAGAGCTTCTTCACTGCTACAGA

AAATACTCGCATTAAGATACTGAAATTAATGTATTGTTGGTACGTGGACGTGCTCATCTA
CTTACAACAGTTAAAGCATATAGTTTTTGTATAGGTATTACCAATTTCTTAATTTCCACA
CACGGACATGTTTTTCAGTTTTGGGATGCTGGTAATCACTCAAGTCTGAACAGTTTCAT
TGCTGTTGGACATCTTTAAAGCTAAAGCACCTTCACTACTGCTAGATATTACACATGCCT
GGGCACCTTGATTGCTACTGGATGCCACATGAGCCAAGGCTTGAAATCCTTCACTCTGC
TGTACACTGCCCAAGTAAGGCACCCAAAGACAAGTCCCTTTACTGGTGATGGACACAT
GTACACTGCAAGCTGGACAATTGAGATGCTTCTTATCCTTCTATGAAAGAAATTATTTTG
GACTTCATGATATAGGCATTCAATATCTGTATTTGAAATCTGTCATGCGAATCATCAACA
GAGTGCTCATTTTATGATAACACTTTGGGTAATATGTATTAACCATCACTTATGTCCCAAA
TAGGTTGTACTACATACATAATTTTTCTGTATGGGACACCAATAACATGAATCTGTGTAT
GACCAAATAAAATTCATGCAGACACTAATAAAATTTTT

Unigene0011463
TGTGGAAATATCTATTACAGAATGCATTTGAGGTAACACCCTTCCATACATAAAATACTA
CACAGTCAATCAATTAGAAATTAACAAAAGGAATGTTAACCTACCTATAACAAATGGAT
GACTTATGAAAATGTGCACTTCATTTTATGCTACCAAAGTGTGAAACGAGAGAGAAAA
ACATCCCACTTATAAAATTGGTGAGTATATTCATTTACTGGGAAGCAAGAATTTTGTAAA
ACTCCATGTGAGAGAGAGAGAGAGAGAGAGAGAATTACTAAGTCAGCTATAAAAACACA
GGATTCAAAATTCTGTTTTCTAAAGTACTGCATTATTTACATTCCCGTAGTTGAAAACAT
TAGGTAATGCCGACTAAAAAAGATTAAAGCTAAGAACAACAGAAGTTTATACAAGCCT
ACATTTCAGGAGGTAGTGCCATCAGTGCACCTCACGTGGTGCACTGTAGGCATCTAAA
AGGTGTTTGCAGTGTCCTTTCGGCCCCTAGCTGCCCCCACCTTTTAGCCTTTTAATTTAC
CTACATTCCCACTTCCTTTCTTCAGTTTTGCTGTCAAACCGCTTCAACTCCTTTTCACTG
TGTTAAGCACTTTTTCGGCGCTTGTCTAATGACAAAAATTAGGATATTCAGCGCCAATA
CGCACCGATTTCCGCTTATCTGCTCCGATAATTGTGTACTGGCACCGATACACATCTAAC
AGAGGCGCCGCTAACCGAAAATCACCGATTTTCAGTTATTGGCAATTTTTGCTTATCATC
ACGCCGTCGGAACAGAACCCCTGCCGATAACCGGGGATGAATGGCCAAAAGTGCCCC
AAGAGTTGGCTTGACTGCCTAAATTTCATAAAAAAATCCTACGAAAAAACTACAAAATG
AAGAGACTAACTGTTTTAAAAGTAGCAAGGTTCCTCTATAAGGCCAACAAAATTATACT
TTACTGGGAATACAGTACACTAAACTGGGAACACCTAAAAACAACAGAGGCATTTCTT
AAAATCAAGCAAATCTAATAACCTGAACAAATACAGGCAGTCCCCCGTTACTGGTGGG
CTCAGTTATCGAGATTTCACAGTATATTGTATATGGTCATCATGGATTTTGTTCCATACTG
AAAGGAAACCTACAACCTTTCTCATTTTTAATGCAATAAGAAGCCATACATAAACACCA
TAAAGGTTAAGTATTTGAGGTTAAACATTTCATGACCCATTATTCCTTTTAAATTTCCAG
TTACATAGTACATACTGGCAACTGAGACAAAGGAAATGACATCCACTTGGCTGTGATAA
TAATTCCATTATAACTTGATTTTACTTTTTTGCTGATAAACCCGTATCCTACGACAAAACC
CACTGTCAATCCAGCTGCCTATTTCAAAATTCACTTTGAAACATAGCTAGCTGGACAAC
CATATGCTACCGATTTTAAATGATTTCACATTTTCAGAAACGTAAGGCTTAGTGGGACCT
CAACTTAACGAAGGGCTAAGGAATCTGCCCTCTGGATAGGCAATGAAATCCGTTTAGTA
ACAAAGATTTACATAACAGCCTACTCGATACTATGGAGCCAAGTTGTATGGCTTATAGA
GTATGGTAATTCTATATAAAGTAATTTATAACCTATCATAACTCATATTTTGCAATTATCA

Unigene0011482
CTTCAGAGGGATACGGCTAAAAAAAAAGTTCAATCTAGAAATTGTCGTAATTATGGTGAA

ACTGAAATACGAGTTGCGGTCTTTTATCTTGAGGATGGACTTCGAATACTTAAGAAGGA
AGAGAAAGAGAGAGAGAGAGAGAGGGGTGGTAATGGAGACCCCAGATAAGTTACAC
CACGTAACTTACACCACATTTTCGATATTAACATAGAAGCAAACCCCCATTCAACAAGC
TGCGATGTTTGCAGGCGGCCTCTTTCTCCTAGATGTAACCCCAGGATCCTTACGTTTAG
CAGCTGTATACGGTGCAGCTCTCGCTCTGACAGTCATAATTTTTGGAGCTCCTGTGGGC
CGATTAGTTGACCGAACTTGTAGGCTTAAGATGGCACAGGTCAGCTTGTGTATTCAAAA
TCTAATGGTCTGCATATGTGCAGCTATATTAGTAGTGGTTTCTTCTATGCTTTTGAGCTCA
ATCTCTCTTCAGAAGCCGCTGGCCGAGGAAGACGCATCTCTTGGGAACAAATCTGATA
AAATCAACCAAAGATGGGTGACTCTCAGAATGATGAGACTGAAGCTAAGAATTTAAAA
CATGGCCAGGCAACAATCTCGACCAGCGAGCCTCAAGAATCAATAAGCAGTTCGAATT
CCTTACAGAATGTGGAAGGAAATGAAGAATCGTCGTCAAACAGGCCGGACGGAATCAT
TTTACTTCAAGATCACCAAGATGGCGTACCAGAGGCTTCATCGCCCGGCTCGAAGTCC
AATCTTAGCTGGTATGAATAGTGTGTTGAGGTCCCTGGATCTCAGCACTGAGGTTCTTG
CTCCTGTGATTGTAGGCACAATCATGAGCAATGTAGGAAATGCTGCTGGAGGTATTGTT
ATTGCATCTTGGAATGTGGGATCTCTTGTTGTAGAATATGGACTGCTTCACTATCTGTAT
CATTCCAGTGAAGACCTCCAGAAGCCAAAGCTAATTAGTAATATTGGTAAGGAAATGG
AGGGGAATGATTGTGAGGTGGTAGATCCTGTTAGAAGTTCAAAGTGGGATGCTCTGAA
AAATCGTCTCGTCATAACTTATGATACAGCGTCTCTCCCAAGTTCAGTGAACAACTCTA
TTATTGTATATGATCAAAATTTGTTTAGAAAATTTATGGGATTAAAATTTGTAGGAATAAT
GTTTTACCAGGTCAACATTCAGAAAAAGTGCCATTTTAGTGCAAAGTGAAGCCCATCA
GTCAGGGTGATCTCCTTAGGTTGCTTTCGATACTTGAAACACTAGTTCAAATGTGTAGT
ATTGATTGGTCAACTCTTTAAAAGTCATTCTCCATTTGTCAATCATTCTAATGGGTTAAC
TGTTCCCCCTACCCATTGTTGTTTTAACTTTAATAACTATATAACTAATTTTCAATGGTGT
TTCCTAAGAGATTCAGCATGGCTTTGGTGAACTTATTAGGCACATGAAGCTATTCCTCG
GCTTACTAGTTCCCAGGTTTAACATTGCATGAACTGCAAAATGTTGTGTAATGAAGTGT
CACAGAGAAATCTACGTTTGAAGGAGAATATGAAACTAGTGGAGAGGTGGAGACTGA
CTTTACTTCTGTTATTCTTCTTTTAATTGGGATCATCACATCAAGATCAGGACTGTGGAT
AGCTGACTTGTCTGTGACACAGCTGCTCCATGATAGACAAATTGCCAAAAGCACTGTT
GGTCACAGACTCGGCTAAAGAGTTAGCAGATACATGATTGGGACTTTCGTAAAGGTGA
ATAAGACAGAGGCAGGCAGGGAACGTAGTGCTATTGATGAAGTAAACCCGTCGAGATG
CGCAGGCTGCCTCAGGGTGGTCAAGACAGCTCGTTTCCAGGTGTACCTCTCTCACTTC
CTTTCGTCATGGGGGGACAGAATGTGGACAAGTCGGCGAGACTGGTTTAGAGCTTGGG
ATGGCTGGGCTCTGATTGTTGCAGAGGGATTTGTGATATTAACTGCTTGTATTGCTCAAG
TTGGAACACTGGGCTCCAAGTTAACCATTGAAAAGGACTGGATTCTTGTCATATGTGG
ACAAGATCAAACTCCACTTGATAAAGAGCACAGCATATCCAGTTAATGGTACCAGCTAT
GATAGGACCTCCCTGCTAGGAACCAGCAAGGAGAGATAAAGTTAAGGTAAATGTAGCC
ACCAGGAATCCAGCATTTCAAGATATGAGGCTTACTCCTAGTCATTATGGGGAAGAACC
AGACAGACCTCACCTTTCGGGAGAAGGAGAAATGGAAAAATTGCCGATTAGGACCAA
GCCCAAAAATTATAGTAGACACAGATGGAGGGTGACTTATTTTTACCCCAGGCACAGC
AACAGAAGTTGAAACCCCAAGGAGGAATGCATACATTAGCAGTATGTTTAATTTTCCAG
TAAGAGAAGAAGGTCAAGAAAATGTTGAAGAACAACAAATATCTGCATGGGGTGCTT
GGCTTGCCTACATGAGACATCCAGTCAGGGATGCTGGCCTTGGTCTTTCGCTCTTGTAT
ATGACTGTCCTTGCATTTGATAATTATTCCAGAGGTTTTGTGTTTGAATCTGGTGTATCG
GAAACTATTCTTGGTATTCTGACTGCAGTAGCTTCGCTCTTTGGAATATTTGGCGCTTTA

ATGTACCCCATACTAAGAGCCAAGTTTGGTTTGGCAACAACAGGCCTCATTGGTTTTGG
TTCAGAAACATGCTGCTTGGTTCTCTGCGTTGCGTCGGTCTTTGCTCCCGGCAGCCCCT
TTGATGCGTCGGTGGCTATCTAAACAATGAAGCTACTACAGTTGTATAAATACCTTCTAC
TTAACTGAGTAACATTAGTTTGGTTGTTTAGTTTAAATAGTACTTGATATATGTTTTGTAA
TGACAGCTGTCAGGTGACAGTAAAGCATAGGTAATTTGTAAGTTAAATTGCAATGCTCT
TTTTTTAAAGTTACTTTATGTATGCAAAGGTTAAAAAAAACTGTTAACTTCATTAGCAAA
TAATGACTTAGCATAAAACTATGTAAGACTATGCTGTGATATATTTCCAGTGCCTCTGCT
TTAATATACAGCAAATCTGCAGATACCCAAAATGAAAGTCCTGTAACTGTCAATCCAGG
AGGAGCTGTTAATGGGGTTCAATCTTCATTAAACCAGTCACTTGGCCTACTTCGTTCAA
TACTCCTTATAATTCTACCCACACGAGCAACATATGGTTTCCTTATAATACTGTCTTTCAT
GTTCGTTACTACAGGGTGGGCGCTGTTTGGTGTTTATGCTCGAAAAGAAATAGTAAAAT
CGTCTTCTGTGAACAAAGAAGTTGAGATGGATGCCATCCTTATGTACATAGGCAGATGA
TCCAGTCTCCTCAAACGCTTAGCCATGCAGTATGAATGGCTTTTATATTGATGATGCTTC
ACAAGATGAAGGCATTGCTAGCTTAGGTGGTGACAAATCAGTTGGCACCAACATCCAG
TAAGTTTCTGTTTGGTTTCATTACTGACAGTAACTTTGATTGAATTAAGTCCAGGGGTAG
AAGAAGAGAAACGAGTAGTCATGGGGGAATCACTTGCCAAATGAACAGGTAGGAGGC
ATTCTCAAGTCTTCCTTCATCTCCAAGTGTAAGGATTCACAATATCTCCAGTGTTTTAGA
AATATACTATACTCCTTGTGGCCTAGGAGATATTTTTGAACTAATAACACATTGCCATTTC
TTAC

>Unigene0011500
CCTAGTTACAGTGTACAAAACAAAGCCCTTTATATACTGAGTATCCTTCTGCACATGTTG
GAATGGCTATAGAACTTAGATAACAATGAGGGTGAGGGTAACCTGGAGATGGGGGTCA
CCATACTTTCTCAAGCACGCCATCCATTCTTCCTTCGGCTGCAAGGATGAAGTGGGTAA
AATGTATCTGATAGGCTGCAGTTTGCATAACTAGGAAAAATACAAGTTACTTTAAAAAC
TGTTCCTAAGGAACAACCATTTGTTATTCACTCCCAAACAAGAACTTGCAATAATGAAG
TGCAAGGTGACTGGCTGGGACCCATTAATTATACCATAATGAACAGCCACCATGGGTCA
TAAGGATAAGATGTCTAGGGACCTGTGGGTGATATCTCTGAAATAGAATGAGGTGAAG
GTGGTCTGAGTATGCCAGACCCCAGCCCTTATGATGTGATAAAGCAGAAATTTCTTACA
AAAGGAACTGGTCCCTTGCCACTAACATTGTAAACTTTCCCAGACAATGCCACCTTGC
TGACAAACAAAAATCTTCCTACACTCTGGCCTTAAGGAGACAGGGATACAAGCTCCTT
GCCTTTCACATATGGAGATTTACCCCTTAAGTAGGGGTTGTCCCTGGTTAAAAAATGGT
GAGTGCTCATGAGGAGAAAAACTACCCATTGTTGCCTTGCAAGCACAGGATGAAAGAT
TGAAACATCTTCCTCTTGTTATAGGAGAGACAGGGCAATGCATCCTAACCTATTAGAAA
AGGTGCTGACAGTTTACCTACCTTAAAACTCATTCAGTTCCATCAAGTACATGGCCCAA
ACGCACTTGAAACTACAGTACAATAGGCCAACTTCTTTTCCTCCATCTCTCTATCTGAC
CTTATGATGAAAGTATTGTTAGTAACGTTACACTCTTGCGTAAATGCGTACAGCCATAAA
CAACCGACCAAGAAACTGCTGTTGTGCTAATAACAACTGAATCGAATAACAGCAGTTT
TGCTTGTACTTCAGCCATTGTATAGTAGTAACCAATTACTGTACCGCAGTTATGTTACAA
ATGATGTTAAGTGGGACAGGACTGATATATTTTTACATTATACCCTTATTCGCTATGGAGA
AAAGGTCAAGGAAATATACTGCTAAATTCGGAAAATGGGAGGTCCTTTTTAGATAGCAC
TTAATGGCACGGACTGGGCAGTAATGTCTTTTCAAGGCCTCCACAACAAAGACTTGAG
GGAAGGGACTGAGAAAGTAGCTCTCATCAGGTCATCATTCCAATACAAAGGAATGAGA
GAGAGAGAGAGAGAGAGAGACTCCTCCGAGTACTAAATGAGATCAGACAAAAAATGTAG

TTCCCAGACCCAACATAGCTAACTCAACTGAGGTAGAGAGAGATCCAAACCTCTCACC
TAAGGCCTGTGATAGAGCTTCACAGCAAAGACAGACAAGGAAATTGGCTATTTGCTGA
ATAAAGTTCCTATTGGAGAGTAACCCCTTCTTCAACATCAGTCATTGAAGATGGCCCAC
TTTCCTTGGTATACAGGCAGTGCTTGAGTTATGAAAATTTGCCTTACGGTAAATCGATTT
TGCGAAGGGATTAGCAATCTGCTGAGGCCAAGGCCAACATGAAAACCTTTTTCAAGGT
TAGGTCTCTATCAGAGGACTGGGACAAGGGCTTGCTCCTACCCCTGTGGTGCAAGGAG
ACAAAAGGGATAGGGCCCTGTGGCAGAGTTTCCCAAAGAAAGGGAACTCCATAAGAA
GCCAATATATCTGGACAACAAAGACAACTCTTGATGAGACGGCCAGTCTGTAGACAGC
ATTTTTTAACTAATTATAAGTGATGGGTTCAGAAAAGGCTTTACCTAAGCTGTCCTAATG
GGGCAGCTGTAAGGCTGTGATGGACCAAGGAAACATCCAATTACAGGAAGGCACAAC
TGCTCCAAGTTTCTCATTAATACTGATATGACAATATTTAGAAAATATTTAATTTTGCGTG
GGCAATCAGGCAGCGAGAAAGATGAAGCTGCAAGTTGTAGCTGAACCTGAGAAAACA
ATGTTCAAGCTGCTAATGACTATAAATTATCGTGCATC

Unigene0011612
ATTTTCACTCGTCGTAAGAAACATGGTTTCTGACTTGTTATGACGAGGGTCTGATCATC
ACAACCTCCTCCTGGGTCGTCTGTGACGAAGTCCCTCAGCCCTACAACATAATCTTTGC
TTTATAAATGTTCTTTCGGAATACTTTAAGATTTTGTCGGAATGGAAAGATCCTGCAGGG
ACCAAGAGTAGACGTTAAAGCGCCTCTTAGAAGTTCTGTCTGGTGTCTGTCATCTGTCT
TCGTCAGTCCTTCACATGACTTCAGTAAATTTGGGACAGGCAATAAAGAAACTTTGGAT
TCACTCCCAAAGGAGAAAGGACGAGGCAAACCCTTTGTGAGAAATTACTCGAGGCTT
TTTCACTTTACTTCGTCACTCGGCATCTTTACAGCTACTAGAGGTTTGAGCTTGTCCAG
CATGGGGGTTAAGGATTATGCAGATGACGTCATTAAGTCCGATGAGGACCAGCGACAG
TATCGTGCTCTCATCCTTGATAATAGAACAGAGAAGTACCCTTGTGAAAATGAATATAA
CAAGTATTTAAATGAGCATGGTGGCTCCAGTAATGCCTACACTGCAGCTGATCACACCA
ATTACTACTTTGATGTGGCTCCAGATATTTGAAGGTTCTTTTGATATCAGACTCAACAAC
TGACAAATCTGCAGCTGCTATTGATGTTCATATTGGCAGTATGTCAGATCCCCGTGAGCT
TCCAGGGTTGGCTCATTTTTGTGAACACATGCTCTTCATGGGCCTTTCCAGGAGCTTTA
GACAGATTTGCACAATTTTATGTATCCCCATTGTTTACGGAGAGTGGAGTTGATCGAGA
GGTCAATGCTGTCAACTCTGAGCATGAAAAGAATGTCCAGAATGATTACTGGCGGCTT
GCTCAACTTGAAAAGTCAACAGCAGAAATAAATGTTCGTGATCATCTCTTGGAGTTTC
ACAACAAGTGGTACTCCTCCAATATCATGTCTTTAGTCGTCCTAGGAAAAGAAAGTTTA
GATGAACTGCAAGGCATGGTGATCGATCTTTTCTCTGGGGTTGTAAATAAGAATGTCAC
AGTACCAGAATGGACAACACATCCTTTTGGCCCAGATCAATGCCGGAAGTTAGGGCAT
GTTGTGCCTGTTAAAGACATCAGAAATCTGTACATCACATTCCCTATACCAGACCTTCAT
CCTCATTACAAAACTGCACCAGGTCATTATCTGGGCCACCTGATTGGTCACGAAGGTCC
TGGTTCATTACTTTCTTACCTGAAGGGCCGTGGTTGGGTCAATTCTCTAGTTGGTGGAC
AGAAGTCAGGAGCAAAGGGTTTTGCATTCTTTGTAGTGAATGTGGACTTAACAGAGGA
AGGCATTGAGCCTAGTAACATTAGGATTGCTGTGGTTGGTAAGGTGTTGGCAGATAAAG
TGACGGAAAAGGAGAAATGGTATGGAACTGAGTACAAGATGGATAGTCTTTGTAAAGA
GCAATGTAGAGGACATTGTCACAGCTGTGTTTCAATATTTGAATTTACTGAAGAATCAA
GGACCTCAAGAATGGGTTTTTAATGAATGCAGAGACTTGAGTGCAATGAATTTCCGATT
CAAAGATAAAGAACGTCCTCAGTCCTATGTTTGCGGGTTGGCAGAGCAGCTCCATTACT
ATCCCTTGAAAGAGGTGTTGTGTGGTGGCTATCTTCTGTCTGAGTATAAACCAGAATTG

ATTGAAAAAGTTCTAGATTGCCTCACTCGTTGGATCTCTGGGAATCTGCTGGCTCAAAT
GATCTTCTTCACCTTCCTAACAAGAATGAGTTTGTTCCATCATCATTTGATTTGCTGAAG
GAAGATAAGGGAGTTAGCAGTTTACCAGAAATGGTTATGGAATGTCCTTTAGCAAGGG
TATGGTACAAGCAACCTGTTGCACATGTTTGCGCAGTTATTCAACTGTACCAAGCTTGG
AATCTTTTATCCATCGATTCCTCTCTGGCCTTCACCTTGAAATGCTTATTCATGGAAACA
TGTCCAAGGAAGCTGCTTGTAATCTTGCTGCTGCTGTTCAAGATAATTTAACAAAGAGA
TCCCATACCCGATCACTGCTCCCATCTCAGCTTATAAGACAACGAGAATATCAGCTTAC
GGATGAGTCCAGTTATGTGTACAAAGCTGAAAATCGCATTCATCGGAGCTCTGCTGTGG
AGACTTACTTTCAGTGTGGTGTTCAGGGAACCCATCAGAATATGCTGCTAGAGCTTTTG
TGCCAAATCATTTTCGAGATGATGAATTCAAATTGCCAAAAGCTGTAGTATATATGGAAC
TTTTTAGTCCTTTGGCTTACTTTGATCCACACCACACAAAGCCTGCCTTTGATGAATTG
AGAACGAAGGAACAGCTAGGGTACATTGTGTGGTCTGGAGTTCGTAGAGCAAATGGA
ACTCAAGGTCTGCGTGTTATTGTTCAAGGCGATCGTCACCCTCAGTATCTAGATTCTAG
AATAGAAGCCTTTCTTCATAAAATTGGGGAGCGTTTAGAGAGCATGAGTGAAGAGGAT
TTCATTCGCCACCGAGAAGCTCTAGCTAGCCGGCGTCTAGAGCGGCCCAAGAAGTTGT
CACACCTCACAGCCACATGGTGGGCGGAAATTATAAATAACCAGTACAATTTTGACAG
AGATGTGGTTGAAGTTGGGCATTTGAAAACCTTAAAGAAACAGGATGTTATTGATTTTT
ACAAGTTATGGATCTCATGCAGCCATACATCACTATCTCCAAGACTTCTAAATCGAAATT
GTAACATTTTCTGCAAAAGATTGTGAACTTTGATGTAAGTAATTGTAGGAATTGTGTATT
TACATATAAAAGTAGGTTATGTGTTTCATAAGGAGTTCCTGTAGCTGGGCTTTTGCAGAT
ATGTACATTGCATATCAGAGGAAAAAGGAAAGTGAAAGAAGACTTGTCAGTGTCATAA
TACTGTAATTTTCTGTGTGAGAATTATTTTTAATAGTTTTTTATTGAACCATAATGTTAGA
AACTTTAGTATGGGAGTAATGGTATTGACTGGGGACTGGACCACTAAAATGTCCTACCA
GAAAGTGACCTTCAAACAGTATTCCACCATTATAATATTTAGGATGTTCAGATCTCAGGA
TAACATTCAAATTAGCATAATATGATATATCAACTTGTTTAATTCATTATTTAAAAAACAT
TGGTACATTATATTGCATGAATGCGAAAAATTTTTCAACCTAGTTTGCATGAGGCTGCCA
TCTGCAGTGTCACCTTCAGTTTTGCTGACACAATTTTCTTTCTGGCAGATTATGATACTA
GAATTTTCAGCATTTTGTCAAAAAATCAAACTAGCAGTTACATGCAATATTTATATTGCA
TTTGCTCATTAATAAGCTTAGGGCATTGATTTATATGTTAATTTTTACACTTGTTGTTTCTT
TCCTTTGAGGGGATAAAGTTTGGAGTGAAGATACAATTACAGCACTGTGATCACATAGA
TTTATATGAAAAATATATGTATAGTTTGATACTATTAGGTTATTAATGTAAACATTATTTGC
GATCATAGTGAGGTATAAGGAAATTTAGTTAAGTAATTGTATATCTGTGAGTTTGTAAAT
CCTTACTTGCAAGTATTAGTCTCAACAAAGAGCTGGGTTTGGCTTTCTTTGAAGCAATT
CCAAAAGAAGAAAAGCCATCAAGTGGTTGAAGGTATTTTTCTTCATACAATGAACTAC
AGGGTAATGGTGAATTGTGAAGGGTTTCTTGATTTTATGAATGGTAGGAAGATTGTGTC
TGTGACAGTGACCTTTGTTGAGATTTATAGATAAGTTACAATCAAGGCAACATTGAATAT
TGGAGCATTGTTTAAATCTCAAGTCAAGTGAAATTTTTTTAGTTAATGCTTGCTTGTGTA
ATGTTTCTGGACTTGTAATAATTTTGGCACTGAATGGCCTCCATGGCCCTAGGATTGGG
ACATATTGCCCAAATTTCTTAACTTGTATTAGTTCATAAATTCATTCATGACATGGGTGGT
GGAAGTAAGAAAGGCATGCAGTTTTCCTTATTTCAGATACAGGTATCCTGTCAAGGTTG
CCAGAATGAAAGGGCAGAAAGTACAGTATCCCAACCTAGCCTTGTCTCAGTGACTGAT
TTATATATTAGTCTTGCAAGTCAGGATTTGCATTATTTTGAAATATCTAGAGAAGAGCAC
CCTGCTGAAAAATTGACAGCATTAATGACTTTATCCTTAAGTGCTTCACTTAAATGGTG
CAGAAGTATTATATTGTATCTGTTGCTCCTAAATGAATTAGGTTTTGGATCTGAGTTTATC

AGAGTTGTTCAGCTAATAGTGTTCCATTAAAGCTGTGGTATCTAACCTTTCTTAACTTGA
GTTATATTTTACAAAGTTTTTAAGTATAGAACATGGCAATAACACAAAAATTTATCAATA
TTTTTACCATATTTTAAGTGGCATACATTCATGTAGTATATATGCATATTTGTGAAATAAAT
CTCAAAAATGACATGAATTTATATTCATCATTGCCAGCAAGCCATTTAATGGTTTTTTGT
ACTTTTGAATATCCTTTTTCTATTTATGGTCCTTTTTGCTTGTTAGTTTCAGAGACTCCAA
AACAGTATTATGAATATGCCAATAGGGAATCCTGGAAATAATAAAAGTTTTGCAGCTGT
GTAGCAAAAAATAACAGTCGTATCAAATCACACATGGTCTAATCTAAAAGCAGTTATAT
GTGTGGCTCAGACATTGTGAACAAACCAACTGCATTAGAGAGAGAGAGAGAGAGAGAGA
GAGAGATACCATTGATAGGCCACACATGCAGAGAAGATGAAAGCTAAATCCACAGTCC
ACTCAGCTATAGATTAGTCAAGCCTACTTGTATCAGTACTCTCTCTTACTGGATATACCT
GTACATACAGTAATGGATTTTAAACAAGTAAAAAATGACCCAAAGTTTCTTCGCCTCAG
TCAAGTTTTCTGTACAGTGTATAATGCTGTATGAAACTCTCAGCCGCAGCCCATGAAAC
TTTCAACGCACCTCAGCGCAGAAAGCTAGCCGTCCACGTCCTATCTGTTGCAGCTGGC
GGAGCAGGAGATCCTGAAGCACCTAGTTCAGAAACCACACAGCCTGATGATGGTCTAT
CCCTTCCACCACCACTTAAGGAGTGTGAAACCATTGCAGACATTCCAGCCTTCAAGCA
AAGTCTGGCTCTCTATCCCATACTAAAGCACACAACTTACTTATGTAAGACTTGTTTGA
AGAGAGGTTATCCATGTTATCTTTTGCAAGCACATGGCCTGGTGGTGCGGGATGCCCTA
ACAGAGTACACCTATGCAGCAGAACTAGCAGGACTCACTTACTCCCTTTGCAACACGA
AGAGTGGTTTAACGCTTTCAGTTAAAGGCTACAATGATAAGTTACATGTCTTGCTGGAA
AAGATACTAGAGCGCATGACTTCTTTTGAAGTGGACCCAAAACGCTTTGAAATTCTTAA
GGATGCTTATGTACGATCACTACGGAACTTTGATGCTGACCAGCCTCACATGCATTTAG
TGTACTACACATCTCTCCTTTTAGGAGAACATGGTTGGACCAAGCAAGAGTTGTTACAA
GCTACAGAGGAACTGCCTGTGTTGTTGACACCTATAGCGGTGCCAGATGTACGATCATG
GCCAACTTTAACCATAAATAAAATTAAAACTACTGAGGCTACAGGGCTGCAATTTGGTA
TGTTTTGGTGATTGGAGGGTGGATGACCAACATACTAATTTGCAGCCCTCTAGCCTCAG
TACTTTTTAAGATCTGGGGGCAGACAGAAAAAGTGCGAACAGACAGACAAATAGCCAT
CTCAATATTTTTCTTTTACAGAAAACAAAAATCAGTATATATAGTAAATATGCCGTTACA
CTTGTGGTTAAATGGTACAGACAGTAGTGCAAGGAAAGAACAGCCTATGTAAACATTT
GTGTATATTCAGTTACAATGAAGTTTATTTACTTGAATAATGCAATGCCACACACAGACG
TATGAGGTGGGGAGCTGCAGCCCCCCCAAGATTTGGGCAGAGA

Unigene0011716
ATTACAGTTCGCGAAAACTACTCTTGAGAGATAACGTCGACTCTTGCCAAAAATGAAA
TCAAAGTTAAAAAATTATTTTGGGATAAAAACTGCCTATTATGCAATTGCTTTAACGGCC
ATATGAAAAACCTCGATGTGAACATTATTTTTTTATGACTGAATTTGTGTAATGAAACTT
GTCAATTATTTATATAGCTGGCCACCAACTTCATTTTATTTTATTTTTATGAAACACGAGT
GCGACTCCCATCAATACAGTTCTGGGACTGCTAAGGGCTCTGTACTCCTGGTAATTGAA
GTCGTAATTATTATCCTGGGCGATGGGCTCCAACTCTCGACCATCTCTTATGTGGCGAAC
GCGAACCTTTCTGCCAAGCAGATGAGTGTGCAAAATGACCGCAAAGACGTTGATGCCT
GTCGGGGGCAGGGCGGCTTCCGTGCATTCTCCAACGCAGTGGCCTTCAGTGTTAATGG
TGCATTTTATTTTATCCAATTGGGTGAAGACGTTTTTCTGTGACTTAACAACCACCAACA
ACCCCAAAACACGGAAATCCTCCACAACATACAACATAGAGAGTCCATTGCTCCATAA
GGCAGTTCGCACTGTCCTTTTTCTGTGTACAGAAAATTTTACTGTAATAAAATCTATTTA
AACAGGAAGGGGAACAGGCTATTTATTTTGTTTATGTTCTGTTAAGCAAGGATTTCACA

GCACATGTCAATTTACCTTCATAACTTTATTTCAAATGCTTTCCAATCACAACTGACTCA
TTTGTTCCAGTGCTAAAGCTCTTTCTTACGTGTATTTTTTGGCCAACAGGCTTTTTTCTT
CAGCCGTGCCCGTCGTAGAGTATCGTCAGGAAAGAAAGGGCCAGGACAGACACCATT
GCCAGCAACGACGTCTTCCAGGTGCTCTTCCATGACAGGCATGAAGATGTCCCTGTGA
TTGTAATGGTCCACATCTATTGTTTCTATTGGTTTCGAATTCATCTGGTCATCAAACATGA
CCTCCTCTTCAGTGTCACCGTCTTCCTCTTCCAGTTCCTCTCCCTCGCCACCGTGCCCG
ACCAGGTGGTGGTTCCTTCCACGTGCAGTCTTATTCTTCCTTCTCCGGTTCTTCCTTCGC
CGTTTTCGACAGACATTCTCAGCTACCCAAGGCTCTGTAATATTTGGGTATTCGTAGTCG
AGCTTTTCAACGTCAGGGATTAGACTCTCTCCTCTTCTCCAGCACATGGGGTTGAAGGT
GCCTGTGTGCGTGGCATCTTGGAAATATTCAAAATGATTCTTCCAATCATAGTTCAACA
AGCGCCACTCCAAGGTTCTCCCAGCCAATTCCATTGGTTGTCGGATCTTGATGGGGTTT
GAATTCGGCCAAAGCTCTTGAATGCCAAGGGCATGAAGCACAGTGTTCAGGGAAGGT
TTTGAATGGCAGAAAGACAGGTCAACTTTGGGCCAGTAGAAGAGGAAAGACAGGCAC
ATCTCATCCCTCGTTTTAAATCCTCCTAGAGTGATGGATGTCCTTTCCCTACTGTTGTAG
GTGCATTCTCCGATCAGATGGTCACCCGGATAAAACGGTCCTCTGCCGAGGAGGAATC
TGATGTTTCCAGCTGGGGTCCAGACCGATGCTCATGACTCCAGCGTCGTAGAACCGAA
GCTCAGGTGTGTAAATGATACGAATCCCTGAGTAATCGGTGAAATTGTGTTCATCCGGG
TTGTCGTAGTGGGCCTCCATCATGTAGTACTTGGGACCGTTCGGCGTCAGTGGGTATCC
AGCCTCCGAGGGTAAGGTCAGGCCCTCAGATCCAACGGCCCACGCAACCACCACGTG
GTTGCAGGTATAGATGAGCTGAGTCATGTTCTGCTGGTGGCACTCGTGCCCTGGCCTCT
CCGACAGCTCCTCGAAGGCCACCTCGAGCTCCGGACTGAAATCCGTACACTCGTACAC
AATCATGTGGTGGAGGTAACGCTCGTTGCCCTCGGGATTGAACACTGGCTCGTAGCTG
ATGACGTGATTCTATACTTCTCAATCTATATAAATAACATCACTTTTCATACCCTAAGTAT
GAAATACTCTCAAAACACATGAAAGCGAAATGTACAAAATCAGGCACTGTAATATATAG
CAGATCAGGAGCTACTACTGACAGTAAGGACTGTTGGTGTATGAAGAGTTTGCTCTTG
TGATGAATTATGGGATTCTGCGCTTCTTTCGTCCTTCCACATAGTCTCTCTTCCAGATAA
CCTTCAAACAAGCCTTACCATGAAAGGCTGTGCATACACACACCTCGACTTCTCTCGC
AGCACAGGCCTCTTGAAGATCTTGCACCAGTAGACGGTGTCGTTCTTCTCGCCCACTT
CCACGCCGTTGTTGGTGAGGAGCCACGGCTTGGTGGGCGTGGGAGGCCGATGGTGGT
GGGTCCTCTGCGGGTGGTGGCGGTGGCCCGTGTGGTGGGAAAACGAGGCTGAAAACG
GTGTTTGCTGGGGCACTGCTCCCTAATTCGTTTTTGTTGGGGTGGCCCGTCCGCTCTTG
GAAGGTGTCTGGGAACTTCTTGTGCATTTCCTCTTCAAGGTCTCTCTCCATTTCCTGGA
ACTCCTTCTGGAGCTCGCGGTCAGGGGCCGCTGAGTGACCAGTGTCTACGCCGTAGAT
GTCAATGATGCGACGAGGGCGACTGGGCGGGGTATGGGGTATGCGACGGGAATGGGA
AATGCCCAGGGAAGTGAGAAGAAGAAGAAGAAGAAGAAGAAGAAGAAGAAGATGAT
GATGACGAGGAAGAAGCTGCCACAGAGGATGAGGGTTCGTGGGGAGCGGCTCCCCCC
TGGGGGTACTGGTTGCTCGGTGGGAGCTTGTGACCCCTCTCCAGCAGGAACATGGAGC
GAGCCCCCCTTCTGCTGCCCCCGTGGTAGTGGAGGCGTGGTCTGGGGCTTTCCGGATC
GACGGGGTCGTCTGGGTGGTACGCCCACAGCACTCGTATTGTGTCGTTCGATATGATGT
GATCTTACGACGAACGCCCCCGTCTCCTCCAGCCACGCCTCGTCCGTCCACTGCTCGTA
GGACGTGAGGCGCTGCGTGATGAAGGACGACGAGGCGAAGGAGGACGCGTTGGGGA
GCAGCGACAGGAGGACCACCGTCTGCAAGATGGGGGTTCGAGACCGAGGAGGGACT
GACGTTTTCCATAACTGTTAGCATGAATGGCTGCTGCTGACGGCGACCACCTCTGAAG
GGCTCGTGAGAGACACTGCTGGTGCTATCTACTACTTTTTCGGTTCCTTGCCCGACCGG

TCATTCTTCTCCACCTCCTCTAGCCCTTCGCCCGTTTCACAAATCTTGGAATGAAACTAT
TCAACTCTCTCTCTCTCTCTATCTCTCTTTCTCTCTCTCTCTTTACCACTGTACACCGGA
GGCGTAGGATACTTACAGGCGCTTTGAAGGACGTCACGGTTGTTAGGTTCCAGAGGCT
GCTGCTACGTAGCCAGAGCCGCACCTTGGATGTGAGAGGTATGTACACTGCTTCTACTC
CGGCTCCTGCTCCGGCTCCGGTTTCTGCTCCGGCTCCGGCTCCGGCTCCGGCACCTCCT
CCCGCTGGAACATTTCGAGGCAAAGGCGGTGACTGTGTTGGTGGCGACTGCATGGCTC
CTCCACCGCCACGGTGTCTCTCCTCCGCGGCCCGGAAGTTGGTGTGGGAGGAGTGGT
GGTGGTGGTGGCGGTGGTGGTGGTGATGGTGGTGGTGGTGGAGGTGGTGGTGACGAC
GGTGGCGGTGATTACACGAAAAGCAACCGGTGGCGTTTGCGAGGAACAACAATCAAG
GAAGCGTCTCTCAACGGCGAACTCAACGCAGACAAAACAATACTACACCCTTCCAGA
AGAAACTTCAAAGAACTGTATAAAAAATAAACCTCTGACATACAAAACTCTGATATATG
TCACACCATTCACTTATTGGCCAGCCAGCCTCCCGGGGTCTGACGGAGGACCCTCGAT
AGCATCGGGAACCGGGAACTTCCAGCAGCCCTCCTCCTCCTCCTGTTCCTTCAGGAGA
TCTCGTCCGAGTTTAGATTTATACACTCTAGGGACCCGCGAAGGATCTCTATATCTCTCT
CTTGATGACCTTAATCTCCCGGGGCTTGTCGCAGGTGTTGTAGGGCCGAGACATGATCA
GAACGGTGTGGGTGTCGTTTTCGTACCCGCCCACCAGCATCCAGTCCCGTTGCTCGTC
CACAGAGGGCTCTTCGTTACCTTTGCCGTGACGATCTTGCAGGTAAGCCTTCCCCGAG
TGGATAAGTAGAGCCAAGGAACGGATCAATGGCCCCCGGATCGTCGATGTGTGCAAAA
ACCAAACAGACTTAGAAAATAGGAACTATTCAGTCGGGGTGCGAACCACAAACACCAT
TATCAACAAGGGGGTAGGATGCAAGGACGCCCAGTTAATGACGTCACAACTATAGGAT
GTTTCCTCCTGAGTAGGATGTGGTAGTCCAGGATATTTCAGGATAGCAGGTAACAGTCC
AATGCGCTATCGTAGGAGTAGACTTTGCAATCCTAGGCTTCAGATGATCCTAAATTCGA
GCGACCTTTTGTATAATCTCGACGCCACACAATCTCGCGTTATCGAACTTAAATTTTGTT
GCGTGTGTGATTGTGTGTGTGTGTCTTTTGTTTATTGCCGGGGCCACATCAAAGCCTTC
CCGAGGAAAAGCCACATCACAATGGACCGCCTTCGTTGCAAAGGGCAATAATTGTCTC
TGCCGCTTTATTTCGCTCGCAACGAGGCTGTCGTGAGAGCTTACAGATTCCAATTGTGT
ATGTGTGAGTTTGTACGTTCGGTTGTACGCGTGAAATGCACAACGGGACGATTGCTATT
TAGTTATTTTCACAGCTCAGATTTCTTGTATCACTCGAATATCTTTCAATTTTACTCTAAT
TCAGAACTCAAAAGACAACGACCGTTCACGTCAGAATGAACACCTTATCACTGTGATG
TCACCGAAGTCTCATTTCCTCAACACATTCTTGGTTCAAGTCACATTCTCTAACGGAGT
TTGTAATATTCCCCACCCGACAACAATATCGGCGCCGTCCATCCTGGGACTTGACGAGA
GGCCGAAGCCGATGTATCCCGTGGTTCTCGCCGTCACCCTAAACTCTATGTGTTCTTCC
TTGGGCGTCCACTGCGCTGACATTTAGTTATGCCACGAACCGAAGATATCTATCCCTTG
TCAAATCCGAGGGAGTTGGGTTATTGAACAAAGACTGCAGCCTCGATCAGGCAATGCA
GTTTTCCAAATCGTTCTCGAAGTACCAAAATGGCGGTGCCGCGGAGATCAACAACTGG
GCGTGGTTATCAGCCGATGAAGGTTCCAGCGATCCTTTCACAGAAATCAACTTCCTCCC
GGATAAATATACAAATAATTCCAGCGATCACTCTTACCTACGTAAGGTCCAAAGCACTC
GCGGGCTCTATCTTATTATCTCTGATGTGCAAATCAAGCTTTTAATGGAGTCGCAGCAGT
GGGCCGAGGGATTTCCTCCCCGAGCGGTGAAGACACACAACCTCTC

Unigene0011814
GTGCGAGAGGTCGGGTGTCGTGTGTTTTCCGTTAGCCCTCTTTCAATATTTAGCTTGTG
TCGTTGCCCTTCTGCATCTCTCGTCTCCGGAATAAATGCCCCGGTTGAATCATACGTGTT
CAGAAAGCGATATTTTCCACTTTCGGGTCCGTTTTTTGGTGGAGAGAGAGAGAGAGAG

AACCCACGTCAAAAGCACGGGAGGCAACGCTTTAGTTGAGGCTTTATTAATTTTTGATA
TTGTATGACAACTGTGATTCTTGAACTTGGACCCCCTCCCCCAAGACATAGCTGCTAAC
ACCACCGCCCCCCTCCCCACACCACCACCAGTCCTGTGTGGATAAGGAAAGTGTGCGA
AGGAAGGATAGCTCAGGTATTGTGCGTTGCCTAATAGCTTGAGACCGAAGGTTTGCTG
CAAGCAGTGGTGATGATGGTGGGGAAAGGAAAAATATTCAATATTTAAATACCCCTACC
ACGGTTCTTAATAAACGGGTACTTATTTGGAAAGAAAAGCAATACGGCCGTCGTCGTTG
CACAACCATTTGGTTAGATTGATTTTTTCACCACCACCACCGCCCGCACATTCGATCGA
GCAGAGGGGTGAGAATTTGGAAGTCTACCCCCTCCCCCACCAACCCCTGTCCGTAAAG
TAACCCCTCGGAGAGCAGCAACATCGCAAGCAAGCAAGCAAGCAAGAAATTGCCGAG
TGCCGAGGAACAACCCCCCCCCAAAAAAAAAGAAAAGTGTTTCTCTGACTGTTTTCCA
TCCATGCAAAGAATGAGGCCATGTGTCTAAAGATGTCACCAGATTTCTCAAAATGGGTC
CCCTTACTCCATCTCTGCCTTGCCTTTGGGGGTGTGGCTATAATCGAAGGCAGTACCGA
TCGTCAATTTGGGATCTTTTTTTTTTTATATTTTCACAGGTTTGACATCAGTATAGCCCCC
ACGCGTTGCCTGAAAAACTGACAGGTCCGGGGGGAGGAGTTGGTGGAGGTGATGGAG
GAGGAGGAGGGGGAGGAGG

Unigene0011137
GGCCAGACGAGCAACGAGCAGCGAATGCGGTAATCAGTGTGTCAGTGGCAGTTGAAA
GGGAAGGGTGTCTCCGTCGGATCGACATGGAGTTCCGACACGTGATGTGACGATGGCC
AATGAGGGAAGGCGGTCGGACCGAAAAGCCTCATGGGGAACGAGTGCCTTTCAGTGT
GCCATTATCTTTTCCGGTGTAAGGAAGTGAAATACCCGGACAGAAGAAGGCGATGTCA
GAGGTGTTAATGCGAGAGCTTAGTGTGGAGTATATCAAATCTTCCTTCAAGTTTTCTCC
AATCGTGATTACGTTTCACCGACTGTGATAAGTGTTGGATCTCAAGAAAAACGTTGATT
ACTTTTGCACGAGAGTTTTGAAGTGATTAAGAGGCATATCTTCCATAATCTGCTGTGGT
GAAAGTGGATTAAGAGGAATGTGCAAATTACGTGTGTTTTGTGTTTTCTTCTGTTTTGC
GATGCTAGACATCGGGAGTGAAGTTGTTGCTGTGCTTCTCTTTGGCCTGACTTGATTTG
TTTTGAATCTGTATCGACCGACTCACTGGAAAGACATAAGTCAAATGATAAAAGTACTT
TTGGTCATATGAAAAGTTGGCTGCAGAGAATGTCGTTGAGTAACAGAAAGTAGGAGAG
ATTTGTGAAGAAGTTATCAAGACAAACTTTTCGATCCAAGTCAATTTAGGTTTGGTTTA
TTGACTTCGATACATTTCTCGTCCTTTCTTGTGGGGACGACACCGCTGTGGAACTGACC
CTTCCAACTTACACTCAGGATTAATATTGTTATGAATATAAGTATTGATTAATAATTATTGT
TTATTTGAGTAACCATCTCAGGATCTGTCTCTTGGAAGTGTTTTGTTGTCTCCCCAGTTT
TCAGGGGTAGATTCTTGCCTATACACAGAATCTCTTCTCCATCCATTACCAGTTCTCGTA
AGCGCTGGCTTTCTATTCTAAAGATTTTCTACATTATTCCTGTCGTTGCTGTTACCATTAT
TGCCTCCATTGTTGCGAAAGCAACAGGTTGGGAACAGCGAGTAAACAAAAACGAGAA
AATGACCAAAGAGAAAGTTGGCGCCGTCGCCGAATCATAATCTGTCAAGAAGGAGAA
AGAATTTTTCCGTCACTTGTGAAGCAAATCAAGCTGTTTGTATTAAAATAGAAATGTCT
CGGCGATTTTAGAAACACATCTGGTCATATTTTTCTTTGAGAATTCACCATAATTCCGAC
GTCTTTCTTATTCATTTTGTTGTCCATTTGTTATTGTTATCAGTATGCATGACGTGGAAAG
ATACCGGGGTCCATGCCTCATTGTGGCCAAACCGGGCAATAATGGTGGTGTCAGTCCCT
CTCAAGGTGGAGGAGGAGGAGGAGGAGCAGGAGGAGGAGGACAAGGAGGGGGAAG
AGCTCGTCGACATCCAAGCCAGGATGGGTCACCACACTGTCGTCCTGCAGGAGGAGG
AAAAGCAGCATCAGGTGGTTCGCAGCGGCATCCGGCGTCCGGTGGTACGATGCATCAT
GCCCACAGTCCATCAGAGGCCACATCACCACCATCAACACTTGCAGCATTCGTCTTCC

AATGCCCATCATAATCACCATAGTGGCCATTCACATCATCCTCAGCAACACCAATCAAA
GAGCCAGCCTGATGGTTGTGGAGTTGATGGTGAAGAGATGGACGATGTGGAGCTTCTT
CCCTTAGCACAGTTGGCACTCCACACCCACGGCCATCCTCTTATCCTTACAGGAACGAG
AGGAGGGGGTGGAAGTGGATCTTCCATGAGGGGGAGCAGTGCGCCTCCACCTCCAAC
CAGACACATCCAAAACCACCACAGTAGTAATGTTAATAGTAACAGTAATAGTAATAAAG
GCAATCATGGTAATAACAATAACCAACACGTTCAGACAGCGTCAGGTGATTATCATCAC
CATAATCATTGGCCAAGATACCATGGGAACCATAGTGACTTTGAAACCTTATATGCCCAT
GTGTTATAACTACAATGATCCCAAACGGGAAACTGTAAGATTCCACGAGGAAAATAAG
AAAAGTAGTGACCTGGGTCTTGTGCCTTTAAGTGGTGGTGGAGTTGGGTGCGCTTGTG
ACCATTGTGTTGCTGGACAGATGTTCCCAACTCCCCCTCCATCACCTGCATCTTCGGCT
TCATCTTCGTCGTCATCTACTGTCATATCATCATCTTGTTCTTCCTCTTCAGAGGATGAAC
CACTGTATGGCATTGTGAGTGGGGGAGTAGGGTCCTCCAACAGAGAGGCTTCTGGGTA
CGTCATCACCAGACCACCCCAACCCATCAGACAACAGCAGCCCCTGCTGCCAGTGAC
AGTGTGTCCGTGACCTCGGATGAAGGCAGCATAGGTCATGCAGATAACTGCCTGCCTA
GGATTATTAAGCCTCGGAAACGGCGTAAGAAAGATCGCAAGCCAGCAGCAGGGTCTCC
ACCACCTTTGCAAGGTGGAGCTGGGGATGCAGGGGGAGATGGGGATTTCTTGGGGGC
ACATGGAGACTGTGGCAAACAACTTGTCAGATCGCTCAGTGAACCTCAGGCACGGCC
GAGGCTTCACATCTCTTCATCGATCAGCCAATCCTTTGCAGGGCATAGGGATTTAGATAT
ACGTATTGTACGAAGCACTGCCAGGGATGTGCCCCCAATGCGGACAAGGCACTGGTCT
GTGCCTGCAGTTGCAAGGCACCGTCCAAATCAGCAGCACCAGGAGGTGGAGGTCCTT
CTGGCAGAGGGGACAATGCTTATGGTCTGGAGGGTAGTGGAGGGTCACCTCTCACGG
GCATGCCTCTGTTTTCCTTCATGCATCAACGTCGGTCGTCTGGAAGGTCGATCGCTCGA
CCTCAGAATCACTACAGTTACCTGAATCAAGGGGATCCATTCAGTGGATCTTCATTCCC
CTCTTCACCACCCTCCATGTTGGATAGATATTTTATGCATCAGAATCTTGAAGTAACTAC
CCCACATCCACCCGAGAGACCTTGGATCCGTTTAGTGGAGTCTGATTTAACCAAGCCG
TCATGTATATTCACTGTTATGTGTTATAATGTATTGTGTGACAAGTACGCTACGCGGCAA
ATGTACGGTTACTGTCCGTCATGGGCGTTAGAGTGGGAATATAGAAAGAAAGGAATCAT
GGACGAAATAAAACACTATATGGCTGATATCATAACACTTCAGGAAGTGGAAACCGAC
CAGTTCTACAATTTCTTCTTGCCAGAACTTAAAAACGAGGGATATGAAGGGATATTCTC
TCCCAAGTCCCGTGCAAAGCACATGACAGAAAACGAGAGGAAATATGTTGATGGTTGT
GCCATTTTCTGGAGAACAAACAACTGGTGTGGTGGAATTCCTAACAAAAGGGAATGTT
TCAATGGACCATGAAGACTTCAAAGGGTTTGGTTATAAGACTTGTCTCCAGAAAATGG
CCATGGCCAATGCATCTAACCCTTCTTTAGTAAATACAAACTCCAACATCTACACTCACT
CGTTTAGATTATCTGCAGCATATGATTTTTCCATCATGGCATACACAAATTATACGTATGA
TTTGTTTGCCCTGGTTAAAGAGCGCTTAATAGAATTTAACCAACTTGCGATGGCCAATC
ATGACGGTTCAGAGGATATGCTGAACCGCGTAATGACAAAGGACAACATCGGTCTTGC
GGCTTTACTAGAAACAAAAGAAGCAGCCTGGGAAAACTCTGTTCTACCAGACAAGTC
CCAGATCAACCAACACATCCTTGTCTGTACAGCACACATCCATTGGGACCCTGAGTTTA
GAGACGTAAAACTTATTCAGACAATAATGCTGATGAGTGAGATGCGTCAAATAATCACT
CAGATAGTTGAGGAATTCCAACATCGAACCAGGTCTGGCAGGAAATTGGACCCTAATG
CCATTCAGTTACTGCTGTGCGGTGACTTAAATTCTTTACCTGAATTAAAGGAATCATAGA
CTACATCTTCCACAATTCTGACACAATGACAGCCCTAGGTGTCCTGGGCCCAATCGACC
CAGAATGGTTTAGGGAGAATAAGGTTATGGGATGTCCTCACCCCCACATCCCATCAGAT
CATTTCCCACTTCTGGTGCAGCTGGAGATGCGTCCTGCACCATCAACTCTAAACCACCC

TAATGGCCTCCTTCACCGGTAGCTTGGGCATTAGACTTATCTGAATTGGACGTCACTTTC
TCCACATGTGACATATCTAGCCAACTCGATCTCACTGTGTTTTGGTGGGCCCCAGCATT
CCTTGCGATTGAACTATGGTCCTTTTCCCCTTCTTTTATCATCAGCCTTTGATATATTTTAT
TCAAACTTTCCCGATTCTCATGTAATAGAACTTCATTAAAGCCTTGTATATTAAAGGTTG
TACTTTTTTATGGCAAGCATTGTTAATGTAATGCTGAGTACAGCATGTGGACAGATATAA
ATGACGTGGTAGAAATCGCTGTGTCCAACCCTCATATTGTGTGCAGCAGTTGCCCTTAC
ACCTGCCACACCAATGAGGCCTTGTTCATTGTGTTTTATGAGGAGAGCTCACACGTGTG
AGATCAGTGGCAGTTATTTTGGTGTGCATTGGGAAATGATATTTCTGTACAAAATTTAGC
TAATATTTAGGACAGTTTTTAATAAGGGGGTATAATGCAAGAAAAGGTTCTATTATTCTT
GTTTGGCTTCCAAGTATTTGCTCTGGCAATCACTAGAGCATATCCTTTAATGGTCTATAC
AGATTTTTTATTGAATGATAATCTTGCTGAGGTCTCAAGTGTGGAGTTCATTTGGTACAG
TAATCAAGTGTTTGGGCAAATCTTTATTTATTGGTTTCGAACTTCCTTACTGGGTTCTTT
TTTTATCCTCAATCATGAAAATATGGCAACCAATCTCGAAAAGATTTTAGTCTCTAACTG
ACCTCTCTGAAATACAAAATCTTCTCTTTAACAAGACATTGGAGGTGTCTCACCGTATT
TCTTCCTTACAAAGCTATAAACCTGGAAAGACTACTCTCAGAATTATTCATCAAAAATT
AGTTTGAAAGACACGCGAGATTTAGGCTTAAGGATTTCTTGGTCTAAAAACTTCACAG
GGCCCATAAAGCTGTGTCGACCATTATCTATCTAAGTCAATGTACATATTTTTAGGAATG
TATTCAATGAAACATTATTCTGTTAAATTTTGAATCAGAAGTTTTTACTTGGCAGCGTGC
AAAGATTTCCTTGTAAATGATGGCCAATTGTAACATTAGTCCTTGTTTTCCTCCTTACTG
AAACAAATCCATTTTCATGAATCAAAGTCATCATTTAACAAGAACAGTGTTATCCCAGA
ACTGTATACTGTATACAGAAATTTGCAGATTATAAGATTATTA

Unigene0011821
CACAAAAGATTGCATTGATATAATATGCATGTTCTCTGTGGGGTAGACAACAGACCCTA
GATGAATGATAGAGTGCACAAACAGTGAGGCATGGTGGGTTGCTTAGCTGGAAACAA
AGGACCCACTCACAGGTTTCAACAGGAAAGTGTCAGTCACGTACGTTTCACCATCTGA
TAACTCTAGCATTGGAACTGTTTGCTGTGCAAACACTTATGGTGTCATTGGTGTATAAA
CTTCTGAAAAAACTTGAGATTGGTGTTTCTGTGAGAATCTTACATGATTTACACAACAT
TGGCTCTCTGACAATTATGACATAAAGAAACAGAAAGAAATACTTAGTTTCTTTGTTAA
CTGTGATTGAAAGATATGTGTGTAAAATGAAAACAACGAAAGATGGAAAAAGTTCCGA
TGAAGTGGTTTAAGCTACATTCAGGCGTATAGAGAGAGAGATGTGTGCATATTCTTGATAG
CAGTCGACAACAAGATCAAATGACTGAAAACAGTGGAAATATAGAAGTCGACACTAG
ACTATTGCATTAAGTATGAAATTTTGGTCATTTGCTGCTTTAATTTCACTACCCTATAAAT
CTATCACTCTTATGAGACTACAGGTAACAAATCACTAGTCTGGTAATGATTAATCCAGAT
CCTTTACTAATTTAGCTCTAATTCCAGCAAGCATAATTTAAAATTTCCCAGGTCAGTGCA
CCAATCTGCCACAACCATTTGGAGCAAGATCAAATGACTGAAAACAGTGGAAATAAAG
GAAAAAGAAACCAGCAAAATCACTAATCCAGCAGCCTACTACTTCCAGTTAGTGAGAA
ACAACCTGTATTCTGTTAAGTACAAAATATTTTTATTACTCACTAATGTGGGTTAAAGTG
CATCAGTAAAAATACAATAAAGTCTGCTAAAAAGGTTGTTAAAAAGCACCAGAAATCA
GTAAAAAATACATTAGCTCTTTTAAAAGCATTTCTCCTGACTTTCAGCTAATTTACCAAA
AATGTGGCTGCAGTATAACTGAGAGAGAGAGAGAGAGAGAGAGAATGACTGGCTGTTTC
ATAAGTGTAGCATTACTAGCAAAAAGTGGAAGTTGTACGATATGCAAGGTATGTAAACT
TTATTTCATCCATTTGAACTGTTAGTTGTTGGTTGGTAAATGGACTTGAAATCAGTAAAA
GGATATTGCAGTATAAATGAGAGAGAGAGAGTAATGGTAAACAGGCAGCTCAGCTTAA

GAAAAACAATGGAAATAACAAAAATAACTCTTTGTTGCTACATTAACACTTAAATGGGA
ATTAGCTCATTTCAACTTACTGCCCAGTGATTTTACTAGACTTGGATACATCCAGTAAGT
TGAGG

Unigene0011367
CTAAAAGAGACCTCAGAGGGAGTGTGAACAAGCATCTCCAGTGCCATTCCACGAGCG
AATGGTATACCTGAAGAGGCGAAGGAGACTTACTGACGAAGAAGAAGAAGAAGAAG
AAGAAGAAGAGGAAGAAAAAGAGGAGGAGGAGGCAGAAGGCTTTCGAGAATATACA
GCAACTATCGCATACAATGCTCAGTTTGATTTCACTTGTGCTCTCGTCTGTCCTGCTGAC
GATTTCTGCAGATGGAGCCACGCTCGAGGCCATCAATACCGGCCCTCTGTATTACTCGC
ACGTCGTCAAGGGCGGCAGAGCGGAAATGCCCTGCGACATCAGCACAGCGGATCCGA
CGGACAGGGTTGGAAGGAAATTCTCCCTTGAAAAGAAAGACATACCGCATTTCCAACC
AAGCCACTGTTTCTGTTTCGTGAGTAGGGGGTCTTCGGATTGTCCTGATGGGTTGGGA
GAGAGATTGGGAGGGGAGCGAGTCTCTAGATGGGGGAACGCGAAAGATGGAGGACG
AGGGACGACCCTTGAAGTTGGAGCTCCCTCCCACCTTAGACATCATTTCGAAAGAGGC
GTAAACATGAGAACTTTCCTGTTTGCGAGGGCGATGGACTCTAAAGGCCGTGTACGCC
GGTGAGGGAACAACGCCTTGAGATGAGATGTGAGAAATGTGATGATGGAGACTCTGG
CTGTAAAGGAGTAAAATGCGCACCCCGATAATGAAAAAGAAATAAACATAAATATAAA
ACTTGGGAGAGCGGGTTGAGAGCCGTCAGGCAAACACGTTAAACGGCGCACGTTGAA
AATAACAGAAAAAAAAAAATTAGGCACACAGAAATCATGATGGATCGTCGGCTACTAAT
CCATTTTTCCTTCAAACGCAAACGAAAAAAACAGGATAAATCATCGAGGATTTTAAATG
TTTCGACTGTAAAGCTTCCGATGTAGAATAACAAACTCGTGCTGTGGTACAAGGACGG
TATTGGCACGCCTATCTATAGCTACGACTCACGAAGCGACACCCGCTCGGTGAAGGTCA
GGAACTGGTCGGACGAGAAGACCCTCGGAGACACTTGCACGCGACGCTTACATGCAA
AGCTTCCAACACCAACTACAGCGACCCGGTCTCTACTTCCATCGCCATAGAGATGAACT
TCAAGCCCCTGTCGGTGAAGATCCTCGGGTCACTGGAGCCTCTTTCGTCCGGCAAGGA
GTACGAACTGGTGTGCCAAAGCGTAGGGGCACGTCCGGCTGCCTCGCTCACCTGGTG
GCTGGATGGGGTCCAGTTAAACAACGCTACTACCTCAACAGCCAGTTCGGGCAACGTG
ACCCTGAGCACGCTGACCTTCGTGCCCAACGAAAACGACGGAGGGAAGTACCTGAAG
TGCCAAGCGGAAACTCGTGGACGACACGAGCGAAAGCACTCGTCTGCACAGCGTCTC
CAACACGCTCAAGGTTGAGGGTCTCGCCAAGACGATGTCTACTTTGAGTGCAACGTGC
GAGCTAATCCTTGGGTGTACAAGGTCGTCTGGTATCATAATGGGGTGCAAGTCCAGCAC
AACGTCTCCGGCGGAGTCATAGTCAGCAACCAGAGCTTAGTGATCCAGCGAGTTCGTC
GCCAGCAAGCGGGATTGTACACCTGCGTGGCGTCTAACATCGAGGGCGATGGCCAGA
GCAACGCTGTCATACTCCAAGTTCAGTATGCACCTGTGTGCGCTGAAGAACAGACCCA
TGTCTACGGCGCGGCGAGACACGAGGAGGTCTCAGTGACCTGCCGTCTGGACGCCGT
CCCCCCCGTCGTCCACTTCTACTGGAGGTTCAACAGCAGCGGCGACGTCGTGGACATC
GCCGAGAGTCACGTGGCATCGCAGGGCCTGGAGTCCTCCTTGTCCTACGTCGCCAGGA
CCGAGCTGGACTACGGCACCCTCCTCTGCTGGGGCAGCAACAGCCTCGGCAGGCAGA
GGAAGCCCCGAGACTTACGAGTCGGACACCGGGAGGCTCCTGGTGAACATCACGGAG
ACGTCGACGCCCGACTTCACCATCAGGGGCCTCGAGCCCGGGGCCTCCTACCACATCT
ACGTCTACTCGGCCAACATCAAGGGCATCAGCGACAAAAAGTACCTCCAGGGATACAC
CCTCAGGGACGTCGCCGAGAGGCGGACGGCGCAAGTCCGGCCGCCCCCGGAGGAGAT
GGTCTCCTTCACGCCCATCCTGGCGGTGATCGTGGGCGTGGTGGTGTCTCTGGTCCTG

GTCGCTCTGGTCGCCGTGCTGATCGTCAGCCTCAACAAGAAGATCCGCAAAATGGTGA
GCTTATGTGCGTCTACAAGGTCGTCCCGGCGGGTCCTCCGGATCCCCCGGACAACTGC
AGCCTGACGAACCAGACGACGGAGGCGCTGAGGGTGAAGTGCATGCCCGGGTACGAC
GGAGGCCTGGACCAGAGGTACATCATTACGCGGAGTTGTCTTTCCCCAGAGGCGCTCC
CCAGTACCCCCAGGGCACGATACCCCGACGCAAACCAGAGCCGACTATATACGCCCAG
ATAGACCACGGCCTAGGCCCCGTCCCGGTGTCCATGGGCGTCGTGCCAGTCAGCGGCA
TGGGCGGCAGCGTCGTGGGGACCTTAGGTGGCACCATGGCGTGTAACGTGGGCACCG
GGAGCATGTGCCACACGGCACCCATGGTCAGCAGTGGGTTGTGTAGCAGTCCGACCGC
CATGATGGGCATGAGCGTGGCCGTAGACCCTGCAAACCTCTCCGTTGCGTATACGACCC
CCGCCCCTCCCCCCCACGGCTTCGGGGGGAATCCGCTAACGCCCCCTCCCGGCGACAG
AGGCCACTTCGACACGAAGTCGACCCCGGCGGCGCTGGTGCTAGACAACGTCCGGGC
GACGGACGAAGGACTCTACCGTTGCAGGGTCGACTTCCAGAAGAGTCCCACCAGGAA
CGCGAGGACGAATGTCTCCGTTATAATTCCGCCCAATTCTCCAACCATTTTATCCGAAAA
TGGGCTCCAGTGGTGCCCGAAGAGGACGAGCAACCCACCGCAGAGACGCCGCTCATG
AACAATCACAAAGAGAGCGAAGTGTGACCTTTGGCTGGCGTGAGTCAGACTAAGTAG
CGTCCCAGGAACAAGAACGTGGCTGGTCCCTTGCAGACGTCCCTGGCTGACAACAGG
GATCTGGACGACAAGAATCCTGATCTGATTCCTGCCAATGGAAACGGCGAACCGGAGG
GGAAGAGTCCTACCACGCCGGAAGAGGGCGGATTCGGCAGCGTGCACATCTGCGCGT
CGGTGCCATATCCCAGCCCGGGAAACGTGTATGGAACGTACCCCAGAACTCCTAGGAC
CATGGCGCCCAGCTCTCTAGTCCGGTCTCGTCTCCAGTTCTCTTTCCAAGTGGGTGCTG
AGGAACGCTCGTTCACGCCACGCCCTGCCTGCCTCTGGAGCCCTCGGCCAGACGACTT
CCAGGGCAGGGCGCCTGACGGTTTCCAGAATCGTGGTCACAGCGTTGCGGGAGTGATA
TCCCATCGCGAACGGGACAAGAAGAAGAAGAAGAAGATGAAGAAGAAGAAAAAGTA
GCGCACGCCAATGAGAGTCTCCGGTGATACACCACACGCCCTTAGAGGACCAGTGGCT
TCTGGAGGTCTATTACACGCCTCAGGTTTACTTAGCGATGGGGAGCAATTTGAGCCCGG
AGGACATCAAGGAAGGAGTGTATACGACAGTAGGCCCATACGACGAAGGGGCCACTAT
GACCCTAATATGTAATGTCAAAGGAGGCAAGCCTCCCCCTCAGGTGACCTGGTGGATG
GACAGAGGATGTATAAACATCAGTAAAAAAAAGCGACAAATCACAGGAAGCCATAAA
GGGTACAAATTGAAAAGTTGCTCTCAGTGGAAGCCGTAGTTCCTCTTGATTTATTCTCC
CTAAACAATGAGTCATCAAGTCCAAATCAGCCTCTTTCCCCGTGAATGGTGAAAACAC
TTCCAAACCCACTGGTATTCTTTGTTTTGAGACAAATCAATTCCCTCATTACATATATCA
ACTTTCTCCATAATTTGATTCACTTAAAACGACACATGGATGTGTAAAAGCAAGTGGCT
CTCATGTTTTACCCTGGGATTTATTATTA

Unigene0011368
TAAAACTTTACATTTGAGGTTAATAATAACGTGGTCTGTAATTCATATCCTGTTCCTGAG
GAATTCGAAGTTTTGAAGAAAGAGCCTGAACCATAGGGATACAAGATCCCGTAGAAGC
CAAAGAAGGTCCCCCTTGTGCTCAAATACAGGTGCTTTTTAAACCCTCGTGATGATGGC
TGCTACCGCCCCAAGGAAGGCTCTCATTTTTCTTCATTCCTGTCATCTTTGATGGAAATA
CATTTAACGTTTTGGAACACCCCTTGAAGCCAAGGAAGGTCGGCCCCCTTGTAGGAGT
GTTGTTCGAAATTCGTCTTTTCTCATTCGTCACATTTCAAGAGAGGAAAGTAATCCCCA
TTTCCGCTTTTTTCTTCTTCATCTTCTTCTTCTTCTTCTTCTTCTTCTTCTTCTTCTTCTTC
TTCTTCTTCTCGGCTAGAGAGAGAGAAATTCGTTCAAACAAGCAGCCGTTTCTGTTGCGCT
TTTCTTTTCGGTTTTCAATGCTCATTCGAATCAGTCTTCGCCTTTGCAGGTCATTTTTAAT

TCTGTTTAAAAAAAAAATTGCTGCTGCTGTTCGTACCAGTGGGTTTAAAAAGCACCTGT
ATTTTG

Unigene0011994
AAAAAAAAATAAGATATCCATAGTAGTTTAATTGTATGAGGATTAGAAATTATCCTTAGC
AGAGAAGGGATCATTATTTAAGTTGTATGTTTTTTCTTGTTTATGTAAAAATAAATATTAG
CTTGTTAGTTTAAAGGACGGGCGGTTCGTTCGGGCGTCACGTCGGCGAAGGGGAAAC
TTCTGGAATACCTTCCGTCTCCCCGGGATGGAGTTCGGATTTGCTTGCGTACAAGCAGA
CGATCACCATTTGTACATATAATATATTTGTATAACAAACATAATGTTAGGCAGATAAAAT
TCTACTTTTTTATATTTTCCAACAGAATTGTTGACAGCAGTTCAATGACAGGGCAGCTCT
CGTAGGGACGGCAAGTTTGTCATGCGCGGGTGTGTGTGTGTGTGTGTAAACGGGTGTC
GATCGTCCTGTAAATATCAGCTTTTAGCCTGGAGTCAGTCTTAGGGTAGAACGGACAAG
GGTCGAAGCTTGATCGGCCAAGCAGCTACAGTATTAGGTCTTACAAAGGAATGGGTCG
CGGGGTCTCGTCTCGCCGGGCAGACGCGTTGCCAGTCGTGCCGCGACGGATTTCAGTA
TTGCAGCGGGAACCTCTCGCGGAAGCTCTCTGTCCGTCAGCCAGATGACGAGAATAGT
CTCCAAATTCTGCCGGTTTCAACGGACGTGCTCTTCTGTGCGGCGCCAATCAAAAACG
CTGATATTTCTTCTGTGAGATGGTCCAGTTGACGTCTGGGGTGCGTGTGAGTGTCAGGG
ACTCGTCAGAGACTTGTTCGTAAGCAAGAGACGAAAGTAGGTAATCTACTGAGTATTA
AACCGCGAAAAACGGCCGCCACAGAATGTCCCGGGTCTGACATTTTTGCAGCGGACG
CTTCCACGGGCCAAAATGTGGAACGTCCCGCGAGCGGGGGTGTTTCGCCGCGGGACA
TCTTGTGCGAGACACTGAACGATTCCACAGAATGTTCTGCGCAAAGACATTTCTGCAG
TGTGTTGTGAAACAGAACCTCTCCCTTCGACCGAGAAGGGAACGCGACTCTCGCCGG
ACGGTCGAAGGGACGTCTCTCTCTCTGGGTTAAATGTTGCTGCCGAACATTTCTGCTGC
GAGCGAAGGCTCTGTACGTTCTCGTGCGATGTAAGACGATTACGCCGCGTACTTTTATC
ACAAATTAAAAGCGACTCTTAGACCACAGAAGTTTCCCCTCCGCCGACGTGAACGGA
AGAAGTACCTGTAACGTACCTGCGTCGTCGGGAAGAAGCCCCGTGCTACGTTGAAGGT
GTACGAGAATAGCACCTCGCTCGCATGGAACCGATCCGCCCGTCGCCAGTCGACCTCG
CCGACTGTGTGTCATCGAGAGTTTTAAGCAGTTTGCAGGCCGATTTCAGGTAATAGGCA
CGTGCAACCGCTGGACCAGCCATTAAAGTGAAATTGCAGTACTCTATATCATTTGTAGT
CTCGTGGTAAAAGCCCCCCAGTCAGACTTTCGTAACAGACACCAAAGGTACAAGAGG
CGGGCCGTGTACGTACTCCGTGCTGGCGCGTTCGCCCGGTCGCGATTATCGCTTCTGGT
CGAGCGGACGTTCTCGATCGGGCGAAAAGGCAAGAGCAAAGCTCGTAGCTCGTTGTA
GAGGGAGCTGCCCCGTCGTCGTCTGTCAACGTGCCCCCTTACGCCCTCTTCTTGTTCTT
CGTAACCTTTTCTTCTTCTACTTCTTCTCCTCCTCCTCCTCCTCCTCGTCCTCCTCAGCCT
CCTGAACGTTCCTCCTCTCCTCCATCTCACCCCTCCTCCCTCCCTCTTCTCCGTCGTGTA
GGCAACATTTTTCATTGTGTGTTTACTTGTTAGACAGACTTGACGAACAGGTTATACGA
CAAAGATCGAAGTGGAAGTCGGCGCAGAAATCCCAAACAACATGGCCGAAACTCCCG
CAGTATTTAACGGCAGCGTGACTGTGTGTGTGTGTGTGTGTGTGTCGCGTTCTCGTGCAGC
ACGAAGCGAAAGGAAATTGGCAGCTTGTGGTGCTGGAGAAGCCAGAGTTTCGGAACG
CGATCCGTAGATAATTAGAGCAAAGATTCGAAGCACGTCAGTTGGGGGAAGCAGTTTG
CCAGCAAGACGCGCCGACCGGATTATCAGAAGTATGACGTACCTGTTCGATTTCAAAC
TTCCCGGGCATCAGGGATACCTTTTAGGGCCTAATTTTCCTTCAGGTTTGTCTGTGGCA
ACACAACGCTTCCAAACACTTAGCTAGCTCACGTAGAAAAGAAAGAGAAAGAGAGAA
AGAGTGAGAGAGAGAGATAGAGAGATAGATTAGGATTCGAATTGACAAGGTTTAAAAC

GGTGTGTGTCTCGTGTGAGTACGTGCGACTTGCCGTCGAAGGGCAAGAAGAAGGGGA
GGTCTCGTCTTGCTATCTCAGGTCACGACGGAAAGCAACTTATTAAGATAGAAAGAGA
TCGAGACGCCCAGAAGTCCGTTTGTCCGACCTGTCGGCGGCGCCACCCCGAGAGCAA
CGTAGCACCTTCGGAAGCATTCTGCAAAGTCTTGTCAGTCAGGGAAAGGGTTTGGGAC
GTTGCGTCGAGGCGTCTGGGACGAAGCTCGGTCGGTGGGTCGGCGTTGTCAGTCACTT
CCCTCTTCTTGAGTTTCCCTTCCCGGCGTCGGCGACGGTGGTGGCGTAGAACGGGTAG
ATAGGAGACGCATATTAAAGAGGGCCTCTTTACAGGTTAGTATGGGGATGCGGCGGGG
AGGGGGAGGCAGGTTTGGATGCTTGATAATTATTGTAATGCACTCAG

Unigene0012007
TTTTTTTTCATAAACTACACAGATGTCACTTTTTCTAGCTGAAGTTGTAAGAAGTAATTT
TTTTTTACCAACTTTACACTTTTTATTACTGTACAATTAATACACCTCCAATCACCAATAC
TACTACTACTACTGCTACTACTGTTACTGCTTACAATGATGATCACTATGAAATTCCTGTA
CAACTAAAAAGGTTAAACAATACAATTATAAGTGACAGCTGCTTTTACCATCCAAGTGC
TCATCAAAAGTATAGGTACAGGTAAGGGTGGTACGGGAAACCTACTTTGAGTTGAGCA
TCTCTCCCAAAATATACAGTACAGTAATTAAGTAGAACTTCATTTTGTCTTTCCCTTACC
ATTCTTACACACTGCTAACAATATTCTCCTGGGTCTCCTAAATCATTTAACACCTGATTC
CTTATCACCCCAAAATCTACCACCTGGTGTAAAACAAATGGGTTCATTTGACTACTGAT
TCTTTCAGGGACGTCAATCAACAAAGTCTACCAATTACTTTTTGTGAGAGATTTTAAGT
GCCACAACCATCTACAGAAATATTTAAATTCTAGTCAGAACAACTACAACTACAGCTAT
CTGATCTGCAGCCTCGGTTACTTTTAATGTCCGGAGAAGGCGGAGGATATGTCAGTGCC
CCATTAACTTACAACTTTAAGATCAATAAGACCCAGTGATTCAGATGCAAGAAGCATGG
ATATTGCAGTCATAGACTTACTACTGATAGGTCAGAACAAAGGATCACTGACAGTATTT
GTATTTTGTGCTCTAACATACAAAAGCATGCATGCTAAATACAAAAAAGAATGCATTAT
GAGACTAGAAACAATTATGTCAATATAAAGTCAATTACTGGAAATAGACTTTGGGTGAT
AATTATTGTCATAACTCTAGTAGTAGTAGTAGTAGTAGAAGTAGTATGTAATTAGTGGTAGTG
ATAGGACCTATTAAGTCAAGCTCACTGACTGACTATAAAGCAGTTTATGACAGTGCCAA
ACTTCTCAGTGATACAAGGCTAAGCCTGAAGGGTTTGCTCCATTTTAAGGTACATTTAA
TTTAACACATTACAGCTTCTTAAAATTCAGTAAGTGCCAAAGCTCAAAAGTTGAAAAC
TTCAGCAAAGCACTAAATTCTCAATTATCTTGAATTTCAATATTAACACAATGCCTAACT
ATCAGCTTATACAATTCCACTATATAAAGTGCAACAAAATTAAACCTCGTTACACATTTA
TCGCAAAACGTTAAAATTCTGAAGTCATCACATAACAGTTTGTAATGTACAACCTGGAA
TACTATGCAGAGTTAATGATGACACCCTGATTGAAGACTAAAACAAACAGCTGTTACAA
TACTGGCACTTGTTATCTTATCAATATGCAAATGTACAGAACTACTCAAATTTATTTAAG
GAAAATTTGACTCAAAACTAACATAACATATGAGTAAGGGCTAATGTAGTATTGCACTC
TCCACACAAGAATCAGGAGGGGTGGGCCAAACTTTGTTCATGTCAATTCCCAGAAAAC
ATTACAATCATGGGATGACTGACAACCCAACTAATCTGGTAAATAATTTTGAATGCCTTA
TTCCAAAGCTTTCATAAAATTCAAATTGGACACATAAAATCAGTGATGGCTAGTTTCTT
CCCAACTATACTCTAGTATTTTGTCAGTTTCACTCTATCCAAATATACAATTTCTACAAAG
CAGTAATTTATTACATAAGACATAACTGCTCTATTTTACTAGATTTAGACCAAACCCTTT
GGTCAAGCTGCACTAGGGCTGACAAGATTTTAACACAGTTGTCTAAATTATTACTAGAG
TTAGCACCCAAGTACTGTTCAACTTGCTCAGAAATCAAGTAGAATCTCAAGAAAATAA
GCCCATCATCACAAGATATCCTGAAATAGCAGACTATCACCGTTCTCACAACAACCTGG
GTTAAGCTAAGATGCTTTCATAAACTGAGTTGGTCAAGAAGTGGTGTTAATTACTACTC

TCAAACCTTAACTTATAATGATAGAAAAGAAGTGTAAGTAGCTAAGAAAAATGGTGGC
TTATGTGCAGACAATTAGAAGTTGCAGAGTGATATGCATAAAGTATTTCATATAGCTAGC
CTAACATACCCAACCTTGCTTAGAAGGAAGTACTGCCTGCGATCTAAAGCAACGTCTTA
GTGAAAGGCTCATATTTCGAGATTCTACAGTATAAAGTTAACCAGAACGACTTTTGTCA
TCTCAAACTAAATAAACCTTACAGTTGAGCCCTGCGAATCTAGAAATGGTCTGACACGA
TGTACGTACTCAATTATAAAAAAATATCTTTGATTCAACACAACGTTTTACTAAAGGAGT
ATTCAACATGGAACTGAAACTTTCTTACATGACCTACGAAGCTTAAGATTCCCTTTGTT
ACCCACTCTTATTACAAATTTC

Unigene0012050
TTTTTTTATAATGTGCCCAGTTTGGTTTTGAAATTTAATATTTGTGATGTAATTCATCTTA
AGCTAAAGAAAAAGTTTGACAATCAAGGTTTTTACAAAGCTCTGCTGTAATATTTTTCA
TTGTTGTATGTTGGTGGTATGCATTTCATAATTCCTGCCAGTTAACTGAAACACCTTGGA
CTTTGGAGTGATATTACTTTTATATATATATATTTGTTGTTGTTCTATTATAAAGAAGACAT
GTAATCTCCATGTTATTAGAAAATAGCCATGTTAAAAGCAAAACATCTGAAGAGGAGTC
TGTTTGAACAAAGTTTCATCACTCCAGAATATTTTCAGTTGAAGAGATAGTGTTCCGTT
ATGCAGAGATCTGTTACCATTATTATTGTATCTTAATTATAGGAAGCTCAGTTAAGTACAT
GTAGCATTTACTAACCAAAATTTTAATTTAGGATTTTAGTCGGGTTTGGACAGTGCCAAT
ATCTGGTTTTAATAGGTGTATTTGGTAATTGTTAAAACAGACCTCTTGCATTACGAAGAC
GGAATAATGTAGTCTTTTTTTTTTTTTTTTATTGTGATTATCCACTGAAAAAGCGTGTCTC
TGCATTAATTGTGTTGTACTTGGCAAGTGATTAAGGGCTTTTATGCCAATAATCTAGTCT
GAAAGTGTACAGTACGAATAATCTTGGTTCTGTTTTAGAATGGGAAATCGTCTGGTTTG
GATCCCAGTGAATATGGAGTATATTAATTTCATGAGGAATTATTTGCCATGTTGAGCTTTT
TTGTATATTTTGTTACTTAGCATTTAGAGATTGTCACAGTTGCTGGTGTCTTGTCTACTAA
CTTGATGCTGGATGATATTGTAACTAATTTTCTACATATGTCATTTGATTTGTGCCAATTT
GCTCTGGTTAGTTAATGGTAATGCCTCCATAACTGGCTTTGGCTCTCTCTCTCTCTCTCTCT
CTTTCAGTGAATTATCGAGCATGCTTTTTGTTGTGTATGTTTAAGATAATCAACTGTCAG
ATTCTTTCTCTCACATGGAATCCAGTACCTTATACAAGAATTTTTCTTTTATACAAGAATT
TTTCTTTTTATTTGTGGAAAATCAAAGCAATATTGACAAGTTTAATACAATGAGTTGAGT
GATGTTCAGGGTCAAGACAAAAATTGGAGCCATAGATCATGATAAAGAAATACTAGCA
CCACATCATTGTATAAGAACTGCATTTAGTTGCCATATAAAACATTTGTGGTTGCCATAC
AGTTTGTAATTTATTTCATTTTCGTTAAGCCCTTACAAGTTTATGCCAGTATTTATTTCTTT
CATCAAACAAAAATTTCATAGTAGCTTACCTGAAATTAGCATAATTAAATCCTAAGTAAT
TTTTTATCTTGCTGTAGCCATATGAATTTTCAAAATATGCTAACAACTTGAGCACCAGAC
AGAGGTTGTACTGGAGAAGCTGGTCTTGTTACTTACCGATTGGCTTAATAGGTTATATGC
ATACAACTAAAGTGTTGTACTCAAAGGTTGGAATAAAGTTTCCCATGAACTCATTTCTG
ATTTAGAAATTGATACATTGTATTTACCCTTTGTACAACGAATTCCCTGCCCAAAGCTTT
TTGTGCTGATTTGTGGCAATTTATAATAGGCATATTATTACCCTTGTTATTCTGGAATCGT
TATTGTAAATTGGTTAAAGTTGAAGATAATGTGGTGCAGTCAGGTGTCAAGGTTCAAAG
AAAAGTTTCTTTTATCCAAGTTGAGCATTCTGCAGTTCTTCAAAAATGTAAGCCAAATA
AAACAAAGTTCTTTCATGTGCCATTATAGTTAAAGCTCTCTTGTACTGGATGTATTTAATT
ATTCCTTTTAAAACAATCTGTAAAGCATATGTCTCAGGTCAGGAAATGTTGGGGGTTGG
GGGGCTTTTTTTAAAATATGATCTTTCTTACACACTTGAGTTAGCTAATGCAAGCAATCA
TTCACTGCTTCTGCGTACACCCAGTTCATTCATCCATTTAATTTATAGGTTCATTCACTTC

TTATACTCTTAATCATTGGCAGACTTAGCTCTTATCACTGTCCCAAGCAAACTGCTTTTT
GAATAATAATTCATTCATCTTTCTATACTATGCAGCCTTACTAAGAAAGGATGTTTCATTC
CTCTTTGTGTCCTACATTTAATGCCAGTGCTGAGTGTTTTTCCAGTAAAATGATCTAATAT
AGTGTCTTTGAATTCTTCATTTACCAAGGTTAGTTATGCAGCTGTTTGTTTGGGACAGA
GGAATCCCAGAGTATGCCTTTTTCATGATAATTATAATTTTAACAGGTA

Unigene0011495
TTGGCATGTATGCCTAAAATCTATAATTAATCAATCAATCAATCAATCCTTTAAAGATATT
AAAACTGTACTAATTGCTTGCTGGCCAAGCCAACCAGATCGCCAGTATTACAACATAAC
CAAAAGTACCGTAAACAGGAAGTCGCTACAATTTAACATTTATAGAAAATCCACTTTTA
CTGATCCCACATAGTGGCATTAATACAAGCAGAACACTCGAAAGTGTTAGTAATTTTGG
CAGTTTTAACTAGGTAGGCATCTGTTGCGTTACTTACATTGGCCAGTCGTAGAAGTCAT
TCCGAACAAAATCTATCTGAGTATTTAGCTTTAGGCCTCTTATCTGGCATCTCCTGTACT
TTACAAGTGTTGGCATTCCTCCTTGTGAATGAAATTACCAGTGCTAAATTTAGCTTAAAT
GAAACAGATCCATGTTACAAATAATTTATAAGAGAACTATAAAACATTTTAGATATAGCT
TTCACCATATTCATCATCAACATGAAGTTCCCGCCAACAACGGTACTATTCAAGCACTG
TTGAACCATAAGGATATACCATTAACTCTATGAGTCGATCGAAATATTTGTTAGAAAATC
AGACTGAGTTTAGCAATTCATCTCATCCCCCG

Note: Blue nucleotide sequences stand for repeat motif of SSR.
