# Supplementary material for: Genetic Diversity Analysis of 11 Macrobrachium rosenbergii Germplasms Based on Microsatellite Markers
Source: Animals (Basel). 2026 Jan 15;16(2):270. doi: 10.3390/ani16020270 (PMC12837990; doi:10.3390/ani16020270)
Supplement: Supplementary file 1 [file animals-16-00270-s001.zip › Table S1.pptx]

## Slide 1
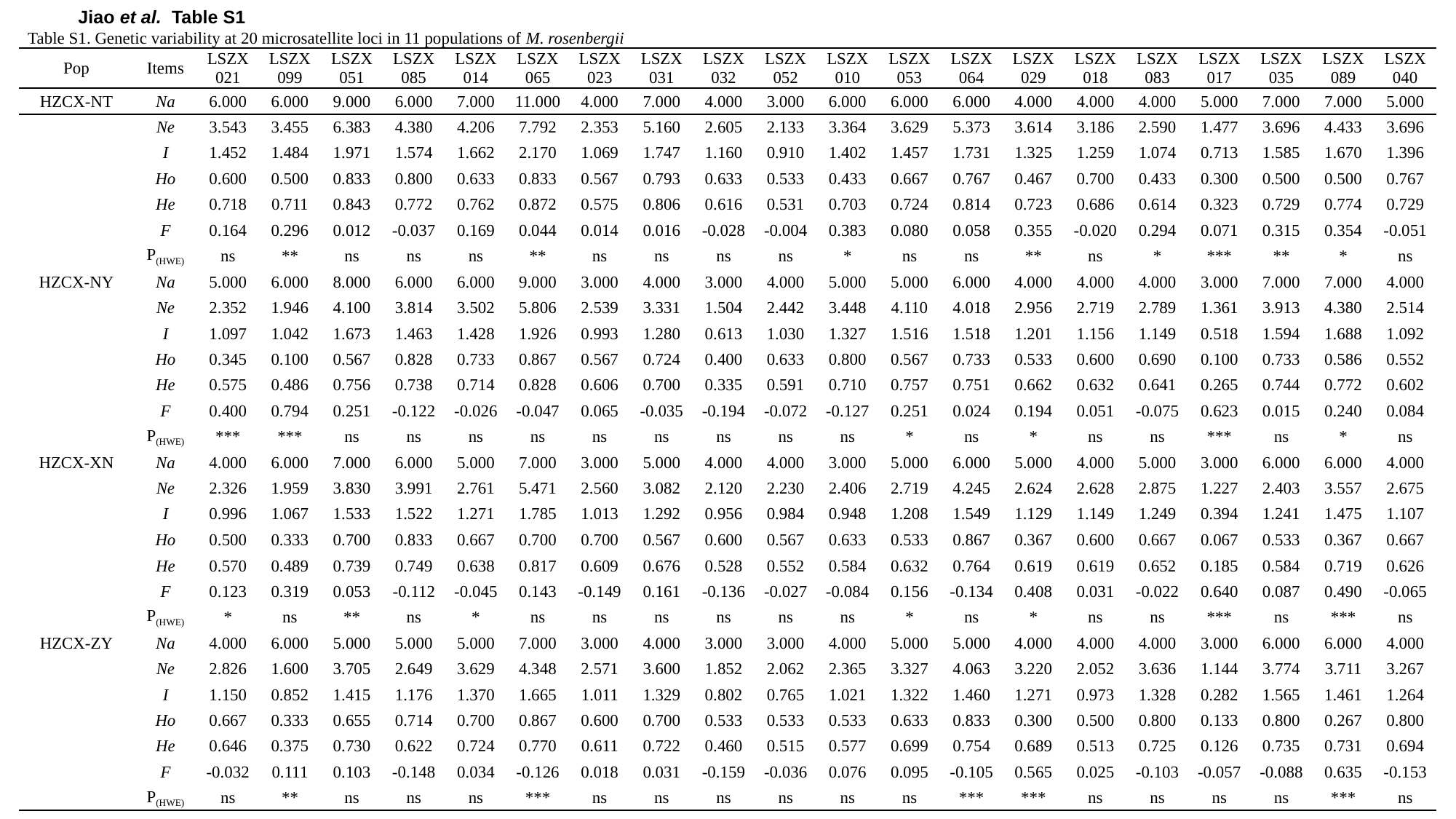

Jiao et al. Table S1
| Table S1. Genetic variability at 20 microsatellite loci in 11 populations of M. rosenbergii | | | | | | | | | | | | | | | | | | | | | |
| --- | --- | --- | --- | --- | --- | --- | --- | --- | --- | --- | --- | --- | --- | --- | --- | --- | --- | --- | --- | --- | --- |
| Pop | Items | LSZX 021 | LSZX 099 | LSZX 051 | LSZX 085 | LSZX 014 | LSZX 065 | LSZX 023 | LSZX 031 | LSZX 032 | LSZX 052 | LSZX 010 | LSZX 053 | LSZX 064 | LSZX 029 | LSZX 018 | LSZX 083 | LSZX 017 | LSZX 035 | LSZX 089 | LSZX 040 |
| HZCX-NT | Na | 6.000 | 6.000 | 9.000 | 6.000 | 7.000 | 11.000 | 4.000 | 7.000 | 4.000 | 3.000 | 6.000 | 6.000 | 6.000 | 4.000 | 4.000 | 4.000 | 5.000 | 7.000 | 7.000 | 5.000 |
| | Ne | 3.543 | 3.455 | 6.383 | 4.380 | 4.206 | 7.792 | 2.353 | 5.160 | 2.605 | 2.133 | 3.364 | 3.629 | 5.373 | 3.614 | 3.186 | 2.590 | 1.477 | 3.696 | 4.433 | 3.696 |
| | I | 1.452 | 1.484 | 1.971 | 1.574 | 1.662 | 2.170 | 1.069 | 1.747 | 1.160 | 0.910 | 1.402 | 1.457 | 1.731 | 1.325 | 1.259 | 1.074 | 0.713 | 1.585 | 1.670 | 1.396 |
| | Ho | 0.600 | 0.500 | 0.833 | 0.800 | 0.633 | 0.833 | 0.567 | 0.793 | 0.633 | 0.533 | 0.433 | 0.667 | 0.767 | 0.467 | 0.700 | 0.433 | 0.300 | 0.500 | 0.500 | 0.767 |
| | He | 0.718 | 0.711 | 0.843 | 0.772 | 0.762 | 0.872 | 0.575 | 0.806 | 0.616 | 0.531 | 0.703 | 0.724 | 0.814 | 0.723 | 0.686 | 0.614 | 0.323 | 0.729 | 0.774 | 0.729 |
| | F | 0.164 | 0.296 | 0.012 | -0.037 | 0.169 | 0.044 | 0.014 | 0.016 | -0.028 | -0.004 | 0.383 | 0.080 | 0.058 | 0.355 | -0.020 | 0.294 | 0.071 | 0.315 | 0.354 | -0.051 |
| | P(HWE) | ns | \*\* | ns | ns | ns | \*\* | ns | ns | ns | ns | \* | ns | ns | \*\* | ns | \* | \*\*\* | \*\* | \* | ns |
| HZCX-NY | Na | 5.000 | 6.000 | 8.000 | 6.000 | 6.000 | 9.000 | 3.000 | 4.000 | 3.000 | 4.000 | 5.000 | 5.000 | 6.000 | 4.000 | 4.000 | 4.000 | 3.000 | 7.000 | 7.000 | 4.000 |
| | Ne | 2.352 | 1.946 | 4.100 | 3.814 | 3.502 | 5.806 | 2.539 | 3.331 | 1.504 | 2.442 | 3.448 | 4.110 | 4.018 | 2.956 | 2.719 | 2.789 | 1.361 | 3.913 | 4.380 | 2.514 |
| | I | 1.097 | 1.042 | 1.673 | 1.463 | 1.428 | 1.926 | 0.993 | 1.280 | 0.613 | 1.030 | 1.327 | 1.516 | 1.518 | 1.201 | 1.156 | 1.149 | 0.518 | 1.594 | 1.688 | 1.092 |
| | Ho | 0.345 | 0.100 | 0.567 | 0.828 | 0.733 | 0.867 | 0.567 | 0.724 | 0.400 | 0.633 | 0.800 | 0.567 | 0.733 | 0.533 | 0.600 | 0.690 | 0.100 | 0.733 | 0.586 | 0.552 |
| | He | 0.575 | 0.486 | 0.756 | 0.738 | 0.714 | 0.828 | 0.606 | 0.700 | 0.335 | 0.591 | 0.710 | 0.757 | 0.751 | 0.662 | 0.632 | 0.641 | 0.265 | 0.744 | 0.772 | 0.602 |
| | F | 0.400 | 0.794 | 0.251 | -0.122 | -0.026 | -0.047 | 0.065 | -0.035 | -0.194 | -0.072 | -0.127 | 0.251 | 0.024 | 0.194 | 0.051 | -0.075 | 0.623 | 0.015 | 0.240 | 0.084 |
| | P(HWE) | \*\*\* | \*\*\* | ns | ns | ns | ns | ns | ns | ns | ns | ns | \* | ns | \* | ns | ns | \*\*\* | ns | \* | ns |
| HZCX-XN | Na | 4.000 | 6.000 | 7.000 | 6.000 | 5.000 | 7.000 | 3.000 | 5.000 | 4.000 | 4.000 | 3.000 | 5.000 | 6.000 | 5.000 | 4.000 | 5.000 | 3.000 | 6.000 | 6.000 | 4.000 |
| | Ne | 2.326 | 1.959 | 3.830 | 3.991 | 2.761 | 5.471 | 2.560 | 3.082 | 2.120 | 2.230 | 2.406 | 2.719 | 4.245 | 2.624 | 2.628 | 2.875 | 1.227 | 2.403 | 3.557 | 2.675 |
| | I | 0.996 | 1.067 | 1.533 | 1.522 | 1.271 | 1.785 | 1.013 | 1.292 | 0.956 | 0.984 | 0.948 | 1.208 | 1.549 | 1.129 | 1.149 | 1.249 | 0.394 | 1.241 | 1.475 | 1.107 |
| | Ho | 0.500 | 0.333 | 0.700 | 0.833 | 0.667 | 0.700 | 0.700 | 0.567 | 0.600 | 0.567 | 0.633 | 0.533 | 0.867 | 0.367 | 0.600 | 0.667 | 0.067 | 0.533 | 0.367 | 0.667 |
| | He | 0.570 | 0.489 | 0.739 | 0.749 | 0.638 | 0.817 | 0.609 | 0.676 | 0.528 | 0.552 | 0.584 | 0.632 | 0.764 | 0.619 | 0.619 | 0.652 | 0.185 | 0.584 | 0.719 | 0.626 |
| | F | 0.123 | 0.319 | 0.053 | -0.112 | -0.045 | 0.143 | -0.149 | 0.161 | -0.136 | -0.027 | -0.084 | 0.156 | -0.134 | 0.408 | 0.031 | -0.022 | 0.640 | 0.087 | 0.490 | -0.065 |
| | P(HWE) | \* | ns | \*\* | ns | \* | ns | ns | ns | ns | ns | ns | \* | ns | \* | ns | ns | \*\*\* | ns | \*\*\* | ns |
| HZCX-ZY | Na | 4.000 | 6.000 | 5.000 | 5.000 | 5.000 | 7.000 | 3.000 | 4.000 | 3.000 | 3.000 | 4.000 | 5.000 | 5.000 | 4.000 | 4.000 | 4.000 | 3.000 | 6.000 | 6.000 | 4.000 |
| | Ne | 2.826 | 1.600 | 3.705 | 2.649 | 3.629 | 4.348 | 2.571 | 3.600 | 1.852 | 2.062 | 2.365 | 3.327 | 4.063 | 3.220 | 2.052 | 3.636 | 1.144 | 3.774 | 3.711 | 3.267 |
| | I | 1.150 | 0.852 | 1.415 | 1.176 | 1.370 | 1.665 | 1.011 | 1.329 | 0.802 | 0.765 | 1.021 | 1.322 | 1.460 | 1.271 | 0.973 | 1.328 | 0.282 | 1.565 | 1.461 | 1.264 |
| | Ho | 0.667 | 0.333 | 0.655 | 0.714 | 0.700 | 0.867 | 0.600 | 0.700 | 0.533 | 0.533 | 0.533 | 0.633 | 0.833 | 0.300 | 0.500 | 0.800 | 0.133 | 0.800 | 0.267 | 0.800 |
| | He | 0.646 | 0.375 | 0.730 | 0.622 | 0.724 | 0.770 | 0.611 | 0.722 | 0.460 | 0.515 | 0.577 | 0.699 | 0.754 | 0.689 | 0.513 | 0.725 | 0.126 | 0.735 | 0.731 | 0.694 |
| | F | -0.032 | 0.111 | 0.103 | -0.148 | 0.034 | -0.126 | 0.018 | 0.031 | -0.159 | -0.036 | 0.076 | 0.095 | -0.105 | 0.565 | 0.025 | -0.103 | -0.057 | -0.088 | 0.635 | -0.153 |
| | P(HWE) | ns | \*\* | ns | ns | ns | \*\*\* | ns | ns | ns | ns | ns | ns | \*\*\* | \*\*\* | ns | ns | ns | ns | \*\*\* | ns |

## Slide 2
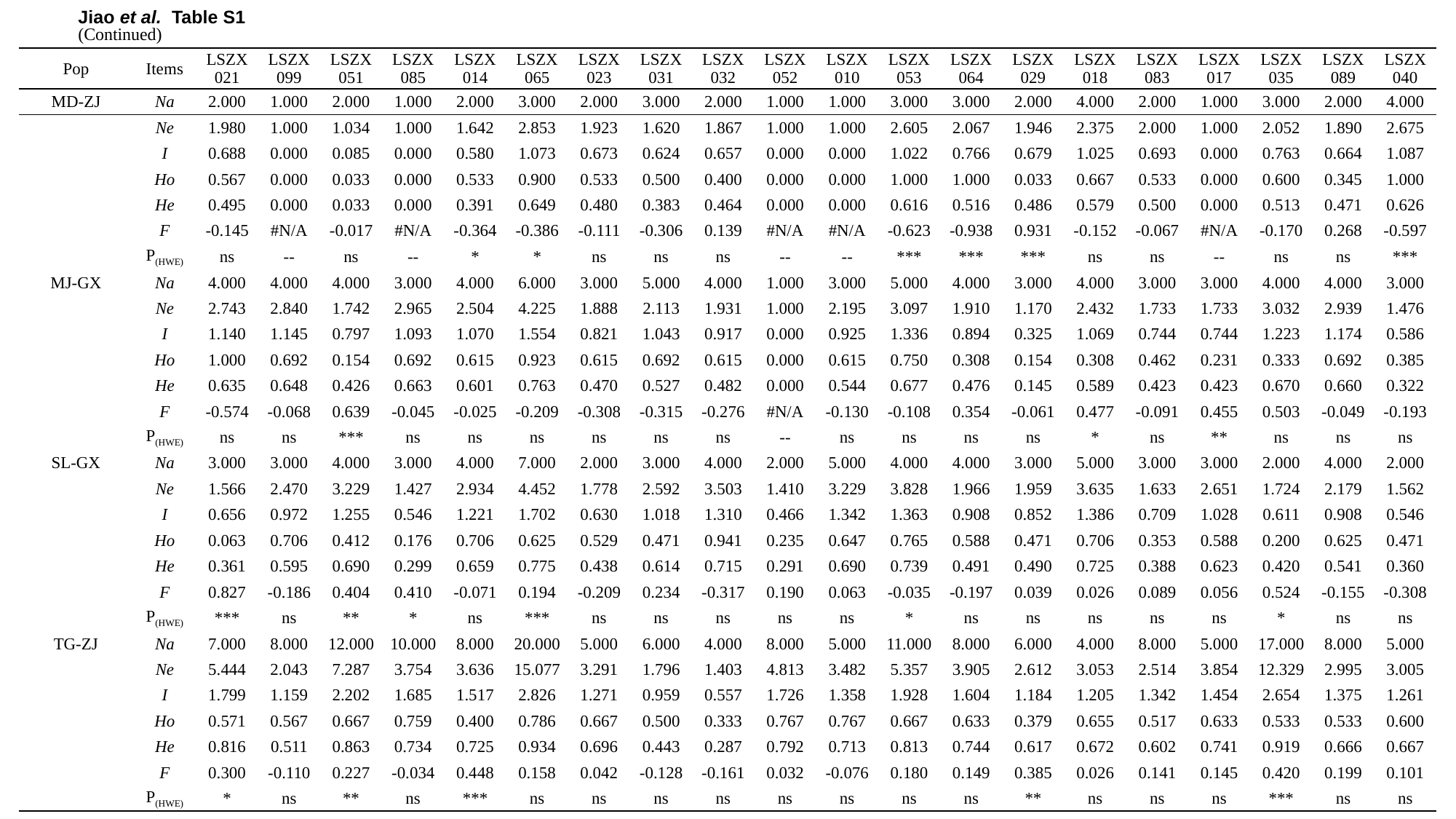

Jiao et al. Table S1
(Continued)
| Pop | Items | LSZX 021 | LSZX 099 | LSZX 051 | LSZX 085 | LSZX 014 | LSZX 065 | LSZX 023 | LSZX 031 | LSZX 032 | LSZX 052 | LSZX 010 | LSZX 053 | LSZX 064 | LSZX 029 | LSZX 018 | LSZX 083 | LSZX 017 | LSZX 035 | LSZX 089 | LSZX 040 |
| --- | --- | --- | --- | --- | --- | --- | --- | --- | --- | --- | --- | --- | --- | --- | --- | --- | --- | --- | --- | --- | --- |
| MD-ZJ | Na | 2.000 | 1.000 | 2.000 | 1.000 | 2.000 | 3.000 | 2.000 | 3.000 | 2.000 | 1.000 | 1.000 | 3.000 | 3.000 | 2.000 | 4.000 | 2.000 | 1.000 | 3.000 | 2.000 | 4.000 |
| | Ne | 1.980 | 1.000 | 1.034 | 1.000 | 1.642 | 2.853 | 1.923 | 1.620 | 1.867 | 1.000 | 1.000 | 2.605 | 2.067 | 1.946 | 2.375 | 2.000 | 1.000 | 2.052 | 1.890 | 2.675 |
| | I | 0.688 | 0.000 | 0.085 | 0.000 | 0.580 | 1.073 | 0.673 | 0.624 | 0.657 | 0.000 | 0.000 | 1.022 | 0.766 | 0.679 | 1.025 | 0.693 | 0.000 | 0.763 | 0.664 | 1.087 |
| | Ho | 0.567 | 0.000 | 0.033 | 0.000 | 0.533 | 0.900 | 0.533 | 0.500 | 0.400 | 0.000 | 0.000 | 1.000 | 1.000 | 0.033 | 0.667 | 0.533 | 0.000 | 0.600 | 0.345 | 1.000 |
| | He | 0.495 | 0.000 | 0.033 | 0.000 | 0.391 | 0.649 | 0.480 | 0.383 | 0.464 | 0.000 | 0.000 | 0.616 | 0.516 | 0.486 | 0.579 | 0.500 | 0.000 | 0.513 | 0.471 | 0.626 |
| | F | -0.145 | #N/A | -0.017 | #N/A | -0.364 | -0.386 | -0.111 | -0.306 | 0.139 | #N/A | #N/A | -0.623 | -0.938 | 0.931 | -0.152 | -0.067 | #N/A | -0.170 | 0.268 | -0.597 |
| | P(HWE) | ns | -- | ns | -- | \* | \* | ns | ns | ns | -- | -- | \*\*\* | \*\*\* | \*\*\* | ns | ns | -- | ns | ns | \*\*\* |
| MJ-GX | Na | 4.000 | 4.000 | 4.000 | 3.000 | 4.000 | 6.000 | 3.000 | 5.000 | 4.000 | 1.000 | 3.000 | 5.000 | 4.000 | 3.000 | 4.000 | 3.000 | 3.000 | 4.000 | 4.000 | 3.000 |
| | Ne | 2.743 | 2.840 | 1.742 | 2.965 | 2.504 | 4.225 | 1.888 | 2.113 | 1.931 | 1.000 | 2.195 | 3.097 | 1.910 | 1.170 | 2.432 | 1.733 | 1.733 | 3.032 | 2.939 | 1.476 |
| | I | 1.140 | 1.145 | 0.797 | 1.093 | 1.070 | 1.554 | 0.821 | 1.043 | 0.917 | 0.000 | 0.925 | 1.336 | 0.894 | 0.325 | 1.069 | 0.744 | 0.744 | 1.223 | 1.174 | 0.586 |
| | Ho | 1.000 | 0.692 | 0.154 | 0.692 | 0.615 | 0.923 | 0.615 | 0.692 | 0.615 | 0.000 | 0.615 | 0.750 | 0.308 | 0.154 | 0.308 | 0.462 | 0.231 | 0.333 | 0.692 | 0.385 |
| | He | 0.635 | 0.648 | 0.426 | 0.663 | 0.601 | 0.763 | 0.470 | 0.527 | 0.482 | 0.000 | 0.544 | 0.677 | 0.476 | 0.145 | 0.589 | 0.423 | 0.423 | 0.670 | 0.660 | 0.322 |
| | F | -0.574 | -0.068 | 0.639 | -0.045 | -0.025 | -0.209 | -0.308 | -0.315 | -0.276 | #N/A | -0.130 | -0.108 | 0.354 | -0.061 | 0.477 | -0.091 | 0.455 | 0.503 | -0.049 | -0.193 |
| | P(HWE) | ns | ns | \*\*\* | ns | ns | ns | ns | ns | ns | -- | ns | ns | ns | ns | \* | ns | \*\* | ns | ns | ns |
| SL-GX | Na | 3.000 | 3.000 | 4.000 | 3.000 | 4.000 | 7.000 | 2.000 | 3.000 | 4.000 | 2.000 | 5.000 | 4.000 | 4.000 | 3.000 | 5.000 | 3.000 | 3.000 | 2.000 | 4.000 | 2.000 |
| | Ne | 1.566 | 2.470 | 3.229 | 1.427 | 2.934 | 4.452 | 1.778 | 2.592 | 3.503 | 1.410 | 3.229 | 3.828 | 1.966 | 1.959 | 3.635 | 1.633 | 2.651 | 1.724 | 2.179 | 1.562 |
| | I | 0.656 | 0.972 | 1.255 | 0.546 | 1.221 | 1.702 | 0.630 | 1.018 | 1.310 | 0.466 | 1.342 | 1.363 | 0.908 | 0.852 | 1.386 | 0.709 | 1.028 | 0.611 | 0.908 | 0.546 |
| | Ho | 0.063 | 0.706 | 0.412 | 0.176 | 0.706 | 0.625 | 0.529 | 0.471 | 0.941 | 0.235 | 0.647 | 0.765 | 0.588 | 0.471 | 0.706 | 0.353 | 0.588 | 0.200 | 0.625 | 0.471 |
| | He | 0.361 | 0.595 | 0.690 | 0.299 | 0.659 | 0.775 | 0.438 | 0.614 | 0.715 | 0.291 | 0.690 | 0.739 | 0.491 | 0.490 | 0.725 | 0.388 | 0.623 | 0.420 | 0.541 | 0.360 |
| | F | 0.827 | -0.186 | 0.404 | 0.410 | -0.071 | 0.194 | -0.209 | 0.234 | -0.317 | 0.190 | 0.063 | -0.035 | -0.197 | 0.039 | 0.026 | 0.089 | 0.056 | 0.524 | -0.155 | -0.308 |
| | P(HWE) | \*\*\* | ns | \*\* | \* | ns | \*\*\* | ns | ns | ns | ns | ns | \* | ns | ns | ns | ns | ns | \* | ns | ns |
| TG-ZJ | Na | 7.000 | 8.000 | 12.000 | 10.000 | 8.000 | 20.000 | 5.000 | 6.000 | 4.000 | 8.000 | 5.000 | 11.000 | 8.000 | 6.000 | 4.000 | 8.000 | 5.000 | 17.000 | 8.000 | 5.000 |
| | Ne | 5.444 | 2.043 | 7.287 | 3.754 | 3.636 | 15.077 | 3.291 | 1.796 | 1.403 | 4.813 | 3.482 | 5.357 | 3.905 | 2.612 | 3.053 | 2.514 | 3.854 | 12.329 | 2.995 | 3.005 |
| | I | 1.799 | 1.159 | 2.202 | 1.685 | 1.517 | 2.826 | 1.271 | 0.959 | 0.557 | 1.726 | 1.358 | 1.928 | 1.604 | 1.184 | 1.205 | 1.342 | 1.454 | 2.654 | 1.375 | 1.261 |
| | Ho | 0.571 | 0.567 | 0.667 | 0.759 | 0.400 | 0.786 | 0.667 | 0.500 | 0.333 | 0.767 | 0.767 | 0.667 | 0.633 | 0.379 | 0.655 | 0.517 | 0.633 | 0.533 | 0.533 | 0.600 |
| | He | 0.816 | 0.511 | 0.863 | 0.734 | 0.725 | 0.934 | 0.696 | 0.443 | 0.287 | 0.792 | 0.713 | 0.813 | 0.744 | 0.617 | 0.672 | 0.602 | 0.741 | 0.919 | 0.666 | 0.667 |
| | F | 0.300 | -0.110 | 0.227 | -0.034 | 0.448 | 0.158 | 0.042 | -0.128 | -0.161 | 0.032 | -0.076 | 0.180 | 0.149 | 0.385 | 0.026 | 0.141 | 0.145 | 0.420 | 0.199 | 0.101 |
| | P(HWE) | \* | ns | \*\* | ns | \*\*\* | ns | ns | ns | ns | ns | ns | ns | ns | \*\* | ns | ns | ns | \*\*\* | ns | ns |

## Slide 3
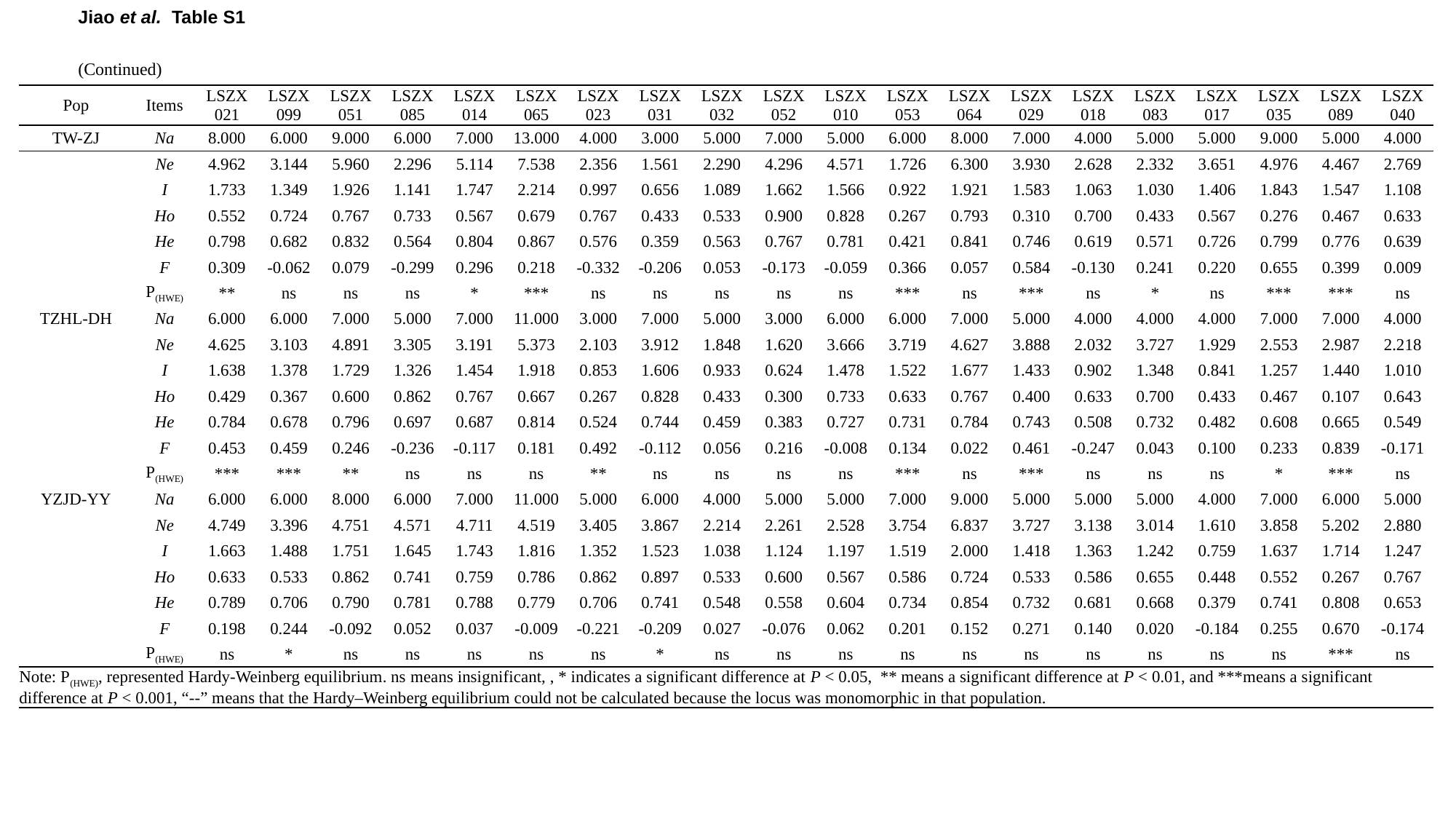

Jiao et al. Table S1
(Continued)
| Pop | Items | LSZX 021 | LSZX 099 | LSZX 051 | LSZX 085 | LSZX 014 | LSZX 065 | LSZX 023 | LSZX 031 | LSZX 032 | LSZX 052 | LSZX 010 | LSZX 053 | LSZX 064 | LSZX 029 | LSZX 018 | LSZX 083 | LSZX 017 | LSZX 035 | LSZX 089 | LSZX 040 |
| --- | --- | --- | --- | --- | --- | --- | --- | --- | --- | --- | --- | --- | --- | --- | --- | --- | --- | --- | --- | --- | --- |
| TW-ZJ | Na | 8.000 | 6.000 | 9.000 | 6.000 | 7.000 | 13.000 | 4.000 | 3.000 | 5.000 | 7.000 | 5.000 | 6.000 | 8.000 | 7.000 | 4.000 | 5.000 | 5.000 | 9.000 | 5.000 | 4.000 |
| | Ne | 4.962 | 3.144 | 5.960 | 2.296 | 5.114 | 7.538 | 2.356 | 1.561 | 2.290 | 4.296 | 4.571 | 1.726 | 6.300 | 3.930 | 2.628 | 2.332 | 3.651 | 4.976 | 4.467 | 2.769 |
| | I | 1.733 | 1.349 | 1.926 | 1.141 | 1.747 | 2.214 | 0.997 | 0.656 | 1.089 | 1.662 | 1.566 | 0.922 | 1.921 | 1.583 | 1.063 | 1.030 | 1.406 | 1.843 | 1.547 | 1.108 |
| | Ho | 0.552 | 0.724 | 0.767 | 0.733 | 0.567 | 0.679 | 0.767 | 0.433 | 0.533 | 0.900 | 0.828 | 0.267 | 0.793 | 0.310 | 0.700 | 0.433 | 0.567 | 0.276 | 0.467 | 0.633 |
| | He | 0.798 | 0.682 | 0.832 | 0.564 | 0.804 | 0.867 | 0.576 | 0.359 | 0.563 | 0.767 | 0.781 | 0.421 | 0.841 | 0.746 | 0.619 | 0.571 | 0.726 | 0.799 | 0.776 | 0.639 |
| | F | 0.309 | -0.062 | 0.079 | -0.299 | 0.296 | 0.218 | -0.332 | -0.206 | 0.053 | -0.173 | -0.059 | 0.366 | 0.057 | 0.584 | -0.130 | 0.241 | 0.220 | 0.655 | 0.399 | 0.009 |
| | P(HWE) | \*\* | ns | ns | ns | \* | \*\*\* | ns | ns | ns | ns | ns | \*\*\* | ns | \*\*\* | ns | \* | ns | \*\*\* | \*\*\* | ns |
| TZHL-DH | Na | 6.000 | 6.000 | 7.000 | 5.000 | 7.000 | 11.000 | 3.000 | 7.000 | 5.000 | 3.000 | 6.000 | 6.000 | 7.000 | 5.000 | 4.000 | 4.000 | 4.000 | 7.000 | 7.000 | 4.000 |
| | Ne | 4.625 | 3.103 | 4.891 | 3.305 | 3.191 | 5.373 | 2.103 | 3.912 | 1.848 | 1.620 | 3.666 | 3.719 | 4.627 | 3.888 | 2.032 | 3.727 | 1.929 | 2.553 | 2.987 | 2.218 |
| | I | 1.638 | 1.378 | 1.729 | 1.326 | 1.454 | 1.918 | 0.853 | 1.606 | 0.933 | 0.624 | 1.478 | 1.522 | 1.677 | 1.433 | 0.902 | 1.348 | 0.841 | 1.257 | 1.440 | 1.010 |
| | Ho | 0.429 | 0.367 | 0.600 | 0.862 | 0.767 | 0.667 | 0.267 | 0.828 | 0.433 | 0.300 | 0.733 | 0.633 | 0.767 | 0.400 | 0.633 | 0.700 | 0.433 | 0.467 | 0.107 | 0.643 |
| | He | 0.784 | 0.678 | 0.796 | 0.697 | 0.687 | 0.814 | 0.524 | 0.744 | 0.459 | 0.383 | 0.727 | 0.731 | 0.784 | 0.743 | 0.508 | 0.732 | 0.482 | 0.608 | 0.665 | 0.549 |
| | F | 0.453 | 0.459 | 0.246 | -0.236 | -0.117 | 0.181 | 0.492 | -0.112 | 0.056 | 0.216 | -0.008 | 0.134 | 0.022 | 0.461 | -0.247 | 0.043 | 0.100 | 0.233 | 0.839 | -0.171 |
| | P(HWE) | \*\*\* | \*\*\* | \*\* | ns | ns | ns | \*\* | ns | ns | ns | ns | \*\*\* | ns | \*\*\* | ns | ns | ns | \* | \*\*\* | ns |
| YZJD-YY | Na | 6.000 | 6.000 | 8.000 | 6.000 | 7.000 | 11.000 | 5.000 | 6.000 | 4.000 | 5.000 | 5.000 | 7.000 | 9.000 | 5.000 | 5.000 | 5.000 | 4.000 | 7.000 | 6.000 | 5.000 |
| | Ne | 4.749 | 3.396 | 4.751 | 4.571 | 4.711 | 4.519 | 3.405 | 3.867 | 2.214 | 2.261 | 2.528 | 3.754 | 6.837 | 3.727 | 3.138 | 3.014 | 1.610 | 3.858 | 5.202 | 2.880 |
| | I | 1.663 | 1.488 | 1.751 | 1.645 | 1.743 | 1.816 | 1.352 | 1.523 | 1.038 | 1.124 | 1.197 | 1.519 | 2.000 | 1.418 | 1.363 | 1.242 | 0.759 | 1.637 | 1.714 | 1.247 |
| | Ho | 0.633 | 0.533 | 0.862 | 0.741 | 0.759 | 0.786 | 0.862 | 0.897 | 0.533 | 0.600 | 0.567 | 0.586 | 0.724 | 0.533 | 0.586 | 0.655 | 0.448 | 0.552 | 0.267 | 0.767 |
| | He | 0.789 | 0.706 | 0.790 | 0.781 | 0.788 | 0.779 | 0.706 | 0.741 | 0.548 | 0.558 | 0.604 | 0.734 | 0.854 | 0.732 | 0.681 | 0.668 | 0.379 | 0.741 | 0.808 | 0.653 |
| | F | 0.198 | 0.244 | -0.092 | 0.052 | 0.037 | -0.009 | -0.221 | -0.209 | 0.027 | -0.076 | 0.062 | 0.201 | 0.152 | 0.271 | 0.140 | 0.020 | -0.184 | 0.255 | 0.670 | -0.174 |
| | P(HWE) | ns | \* | ns | ns | ns | ns | ns | \* | ns | ns | ns | ns | ns | ns | ns | ns | ns | ns | \*\*\* | ns |
| Note: P(HWE), represented Hardy-Weinberg equilibrium. ns means insignificant, , \* indicates a significant difference at P < 0.05, \*\* means a significant difference at P < 0.01, and \*\*\*means a significant difference at P < 0.001, “--” means that the Hardy–Weinberg equilibrium could not be calculated because the locus was monomorphic in that population. | | | | | | | | | | | | | | | | | | | | | |
